# BGAL1 depletion boosts the level of β‐galactosylation of *N*‐ and *O*‐glycans in *N. benthamiana*

**DOI:** 10.1111/pbi.13316

**Published:** 2020-01-11

**Authors:** Ricarda Kriechbaum, Esmaiel Ziaee, Clemens Grünwald‐Gruber, Pierre Buscaill, Renier A. L. van der Hoorn, Alexandra Castilho

**Affiliations:** ^1^ Department of Applied Genetics and Cell Biology University of Natural Resources and Life Sciences Vienna Austria; ^2^ Department of Food Science and Technology College of Agriculture Shiraz University Shiraz Iran; ^3^ Department of Chemistry University of Natural Resources and Life Sciences Vienna Austria; ^4^ The Plant Chemetics Laboratory Department of Plant Sciences University of Oxford Oxford UK

**Keywords:** glyco‐engineering, β‐galactosidases, β1,4‐galactosylation, *Nicotiana benthamiana*, RNAi, CRISPR, Cas9, *Nb*BGAL1

## Abstract

Glyco‐design of proteins is a powerful tool in fundamental studies of structure–function relationship and in obtaining profiles optimized for efficacy of therapeutic glycoproteins. Plants, particularly *Nicotiana benthamiana,* are attractive hosts to produce recombinant glycoproteins, and recent advances in glyco‐engineering facilitate customized *N*‐glycosylation of plant‐derived glycoproteins. However, with exception of monoclonal antibodies, homogenous human‐like β1,4‐galactosylation is very hard to achieve in recombinant glycoproteins. Despite significant efforts to optimize the expression of β1,4‐galactosyltransferase, many plant‐derived glycoproteins still exhibit incomplete processed *N*‐glycans with heterogeneous terminal galactosylation. The most obvious suspects to be involved in trimming terminal galactose residues are β‐galactosidases (BGALs) from the glycosyl hydrolase family GH35. To elucidate the so far uncharacterized mechanisms leading to the trimming of terminal galactose residues from glycans of secreted proteins, we studied a *N. benthamiana* BGAL known to be active in the apoplast (*Nb*BGAL1). Here, we determined the *Nb*BGAL1 subcellular localization, substrate specificity and *in planta* biological activity. We show that *Nb*BGAL1 can remove β1,4‐ and β1,3‐galactose residues on both *N‐* and *O*‐glycans. Transient BGAL1 down‐regulation by RNA interference (RNAi) and *BGAL1* depletion by genome editing drastically reduce β‐galactosidase activity in *N. benthamiana* and increase the amounts of fully galactosylated complex *N*‐glycans on several plant‐produced glycoproteins. Altogether, our data demonstrate that *Nb*BGAL1 acts on galactosylated complex *N*‐glycans of plant‐produced glycoproteins.

## Introduction

Glycosylation is an important protein modification in all eukaryotes, and the impact of different glycan modifications on the function of glycoproteins has been extensively reviewed (Dalziel *et al.*, 2014). *Nicotiana benthamiana* is one of the most widely used host plants to produce therapeutically relevant glycoproteins (Montero‐Morales and Steinkellner, 2018). However, the production of recombinant proteins in plants can lead to the synthesis of aberrant glycosylation that can impair protein biological activity.

Production of recombinant proteins in plants mimicking mammalian glycosylation has been achieved with glycoengineering (Montero‐Morales and Steinkellner, 2018). Modulation of the *N. benthamiana* glycome included the (i) down‐regulation/elimination of glycosyltransferase activity (core xylosyl‐ and fucosyltransferases) to prevent the synthesis of potentially immunogenic epitopes (∆XTFT) (Jansing *et al.*, 2019; Strasser *et al.*, 2008); (ii) down‐regulation of glycosidase activity (HEXO3) to avoid the generation of paucimannosidic *N*‐glycans (Shin *et al.*, 2017); and (iii) introduction of complex human‐type glycosylation such as β1,4‐galactosylation (∆XTFT^GAL^, Strasser *et al.*, 2009), multi‐antennary *N*‐glycans (Nagels *et al.*, 2011) and sialylation (∆XTFT^SIA^, Kallolimath *et al.*, 2016). These glycosylation mutant plants are established as expression host to produce proteins with tailored glycosylation (Montero‐Morales and Steinkellner, 2018). Proteins expressed in ∆XTFT and in ∆XTFT^SIA^ are decorated with glycans carrying up to 90% of terminal *N*‐acetylglucosamine (GlcNAc) and sialic acid (Na) residues, respectively (Castilho *et al.*, 2012, 2013, 2014, 2015; Jez *et al.*, 2013; Kallolimath *et al.*, 2016; Schneider *et al.*, 2013). Despite major achievements, plant glycoengineering must still overcome hurdles such as the correct subcellular localization and expression level of glyco‐modulating enzymes and the adverse activity of endogenous plant glycosidases involved in trimming of complex *N*‐glycans. For example, efficient β1,4‐galactosylation was only achieved upon correctly targeting the human β1,4‐galactosyltransferase (GalT) to the trans‐Golgi cisternae and tightly controlling its expression levels (Kallolimath *et al.*, 2018; Navarre *et al.*, 2017; Schneider *et al.*, 2015; Strasser *et al.*, 2009). Although these approaches succeeded in improving the galactosylation of monoclonal antibodies (mAbs) (Castilho *et al.*, 2011a; Forthal *et al.*, 2010; Kallolimath *et al.*, 2018; Loos *et al.*, 2015; Schneider *et al.*, 2015; Strasser *et al.*, 2009), trimming of terminal β‐galactosyl residues in other recombinant proteins still occurs in the apoplast (Castilho *et al.*, 2014). Therefore, apart from mAbs, studies on the impact of glycosylation on protein function/activity have never included the β1,4‐galactosylated variant (Jez *et al.*, 2013; Kallolimath *et al.*, 2016; Loos *et al.*, 2014; Schneider *et al.*, 2013).

*In planta* galactosylation has been accomplished by transient or stable expression of a late‐Golgi targeted GalT using a chimeric protein consisting of cytoplasmic tail, transmembrane domain and stem (CTS) region of rat α2,6‐sialyltransferase (ST) (^ST^GalT, Strasser *et al.*, 2009). Previous results revealed that approximately 40% of *N*‐glycans on endogenous total soluble proteins (TSPs) are galactosylated, but only 17% of *N*‐glycans are galactosylated on secreted proteins present in the apoplastic fluid (AF) (Schneider *et al.*, 2015).

We hypothesize that glycosyl hydrolases (GH) are responsible for the removal of non‐reducing β‐D‐galactosyl residues. This hydrolytic activity has been reported for β‐galactosidases (BGALs) from different GH families in different organisms (Lombard *et al.*, 2014). β‐Galactosidases have specificity towards β1,3‐, β1,6‐ or β1,4‐galactosidic linkages and are often most active under acidic conditions (Ahn *et al.*, 2007). All plant BGALs belong to the GH35 family. GH35 comprises enzymes with β‐galactosidase activity (EC:3.2.1.23). Many genes encoding putative β‐galactosidases from the GH35 family have been classified based on sequence homology (Ahn *et al.*, 2007; Chandrasekar and van der Hoorn, 2016; Gantulga *et al.*, 2009; Gantulga *et al.*, 2008; Iglesias *et al.*, 2006). The genome of *N. benthamiana* contains 28 putative BGALs, which remain to be characterized (Buscaill *et al.*, 2019). Activity‐based protein profiling (ABPP) on *N. benthamiana* apoplast identified two active BGALs (NbS00024332g0007, *Nb*BGAL1; and NbS00037566g0014, *Nb*BGAL2) (Chandrasekar *et al.*, 2014)*. Nb*BGAL1 shares 74% identity to *Arabidopsis thaliana At*BGAL8 (closest *A. thaliana* homolog) and was mapped as a putative BGAL in the *N. benthamiana* apoplast proteome (Goulet *et al.*, 2010).

Plant BGALs have numerous biological functions. So far, studies of plant BGALs have been focusing on their involvement in physiological processes such as fruit ripening, pollen development and seed germination and in cell wall modifications (Dwevedi and Kayastha, 2010). A recent report demonstrated that secreted *Nb*BGAL1 contributes to immunity against pathogenic bacteria by releasing immunogenic peptides from glycosylated flagellin containing a terminal mVio residue (Buscaill *et al.*, 2019). Importantly, *Nb*BGAL1 null mutants generated by CRISPR/Cas9 (*bgal1)* have drastically reduced β‐galactosidase activity in the AF (Buscaill *et al.*, 2019). Despite the numerous studies on BGAL functions, a direct evidence for the involvement of specific BGAL(s) in trimming *N‐*glycans is still missing.

In this investigation, we cloned the cDNA sequence of *Nb*BGAL1 in different expression vectors and determined its (i) subcellular localization, (ii) enzymatic activity/specificity towards galactosylated glycans and (iii) *in planta* biological activity. Finally, we assessed the impact of suppressing *Nb*BGAL1 activity by transient RNA interference or using *bgal1* null mutants on the generation of recombinant glycoproteins with di‐galactosylated *N‐*glycans.

## Results

We postulated that all recombinant proteins are efficiently galactosylated when expressed in ∆XTFT^GAL^ plants but that apoplast‐resident glycosyl hydrolases, such as β‐galactosidases, subsequently trim terminal β1,4‐galactosyl residues. Two secreted active BGALs (*Nb*BGAL1 and *Nb*BGAL2) were previously identified in the extracellular space of *N. benthamiana* using ABPP (Chandrasekar *et al.*, 2014)*. Nb*BGAL1 was detected with an apparent molecular weight (MW) of 45‐kDa, lower than its theoretical MW for the mature protein (89.7‐KDa), probably indicating that this is a processed protein. *Nb*BGAL1 accumulates in the apoplast and is a functional β‐galactosidase that cleaves galactose from fluorescein di‐beta‐D‐galactopyranoside (FDG) and selectively catalyses hydrolyses of 4‐nitrophenyl‐conjugates of β‐galactose but no other monosaccharide conjugates (Buscaill *et al.*, 2019). Furthermore, null mutants of *N. benthamiana* lacking *NbBGAL1* generated by genome editing (*bgal1*) have substantially reduced apoplastic β‐galactosidase activity as compared to wild‐type (WT) plants (Buscaill *et al.*, 2019). According to these findings, *Nb*BGAL1 is a good candidate to start investigating the involvement of secreted β‐galactosidases in the processing of complex‐type galactosylated *N‐*glycans decorating recombinant proteins produced in *N. benthamiana.*

### *Nb*BGAL1 overexpression and subcellular localization

**Figure 1:**
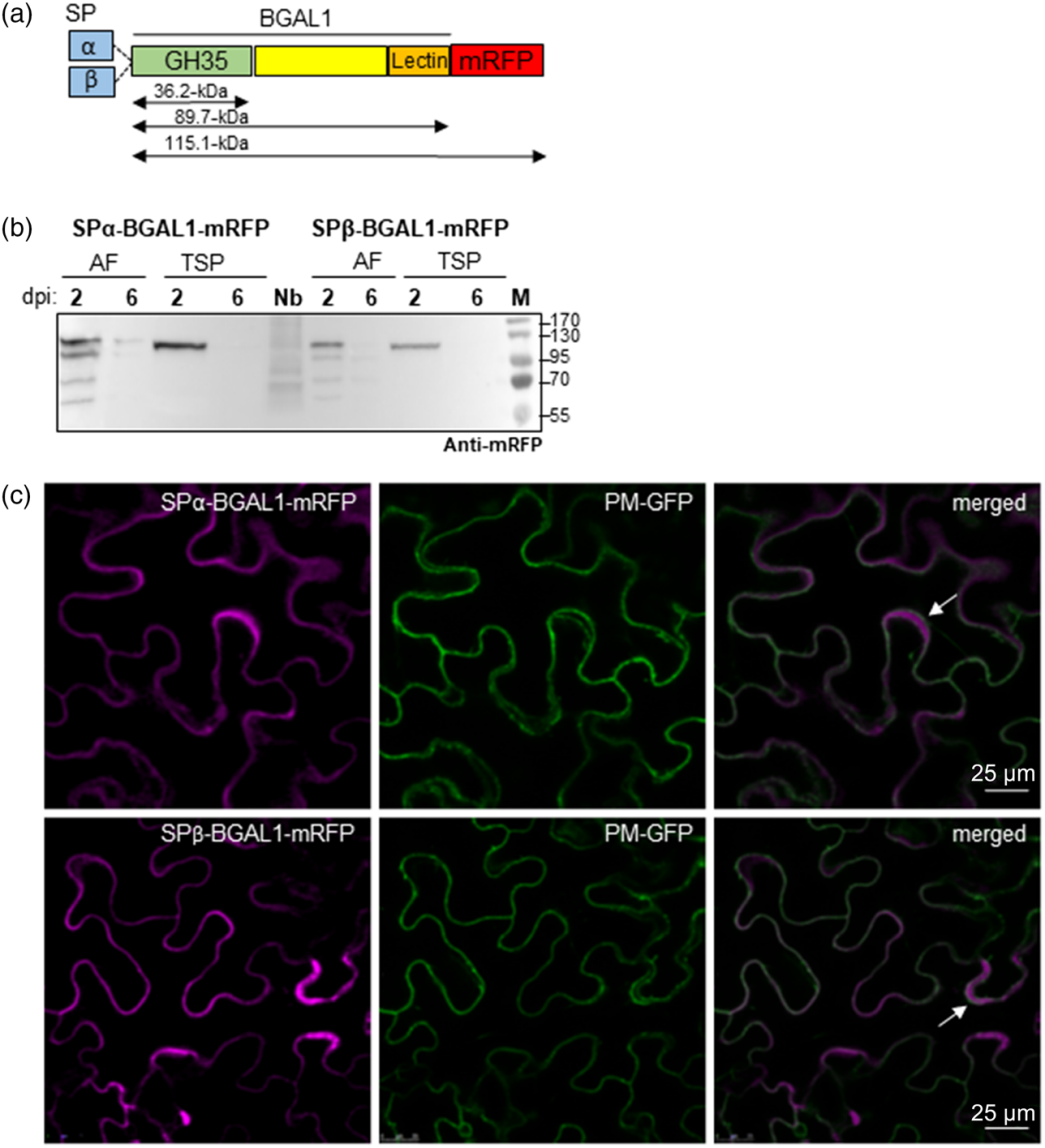
*Nb*BGAL1‐mRFP is a secreted protein. (a) Schematic representation of SP‐αBGAL1 and SPβ‐BGAL1 fused to mRFP (SPα‐BGAL1‐mRFP and SPβ‐BGAL1‐mRFP) showing the different protein features: endogenous signal peptide (SP‐β) or SP from alpha‐amylase (α); glycosyl hydrolase 35 domain (GH35, PS01182), galactose biding lectin domain (PF02140) and mRFP fluorescent tag. Theoretical molecular weights of mature protein without signal peptide (SP) are indicated in kilo Dalton (kDa). (b) Secreted proteins (AF) and total soluble protein (TSP) extracts from ΔXTFT leaves expressing BGAL1‐mRFP were analysed by immunoblotting at 2 and 6 days post‐infiltration (dpi). At 2 dpi, a protein band of approximately 130 kDa is detected by anti‐mRFP antibodies. TSP extracted from non‐infiltrated leaves (Nb) served as negative control. (c) Fluorescence imaging of BGAL1‐mRFP. Subcellular localization of the recombinant enzymes was determined by live‐cell imaging of *Nicotiana benthamiana* leaf epidermal cells expressing SPα‐BGAL1‐mRFP and SPβ‐BGAL1‐mRFP. Fluorescent protein fusions were transiently co‐expressed in ΔXTFT with PM‐GFP, a plasma membrane marker (also partially localized in the ER), and analysed two days post‐infiltration. Merged images show the co‐localization of the fusion proteins with PM‐GFP and also a fluorescence (magenta) signal typical of secreted proteins accumulating in the apoplast (white arrows). Scale bars are indicated.

To confirm the accumulation of *Nb*BGAL1 in the apoplast and investigate other possible subcellular localizations, *Nb*BGAL1 was *C*‐terminally tagged with the monomeric red fluorescent protein (mRFP). cDNA of *Nb*BGAL1 was cloned either including its endogenous signal peptide (SPβ‐BGAL1) or as a chimeric sequence where the signal peptide was substituted by the signal peptide of the barley alpha‐amylase (SPα‐BGAL1) (Figure S1). SPβ‐BGAL1‐mRFP and SPα‐BGAL1‐mRFP fusion proteins consist of the GH35 domain, a Gal‐lectin domain and the fluorescent tag at the C‐terminus, with an expected molecular weight of 115.1‐kDa (Figure 1a). SPβ‐BGAL1‐mRFP and SPα‐BGAL1‐mRFP were transiently expressed in *N. benthamiana* leaves by agroinfiltration in the absence of silencing inhibitor p19. At two days post‐infiltration (dpi), the fusion proteins are stable and detected as a ~ 130‐kDa mRFP‐tagged protein in both AF and TSP (Figure 1b). In AF, several other bands are also detected and most probably represent degradation products. At 6 dpi, the fusion proteins are no longer detected (Figure 1b). The higher MWs of the two mRFP fusion proteins when compared to the calculated MW are most probably due to protein glycosylation (see section on characterization of NbBGAL1). To determine *Nb*BGAL1 subcellular localization, leaf epidermal cells expressing SPβ‐BGAL1‐mRFP and SPα‐BGAL1‐mRFP were analysed by live‐cell confocal microscopy at 2 dpi. The fluorescence signal detected for both fusion proteins is typical of secreted proteins (Shin *et al.*, 2017) (Figure 1c), confirming that *Nb*BGAL1 is an apoplastic beta‐galactosidase.

**Figure 2:**
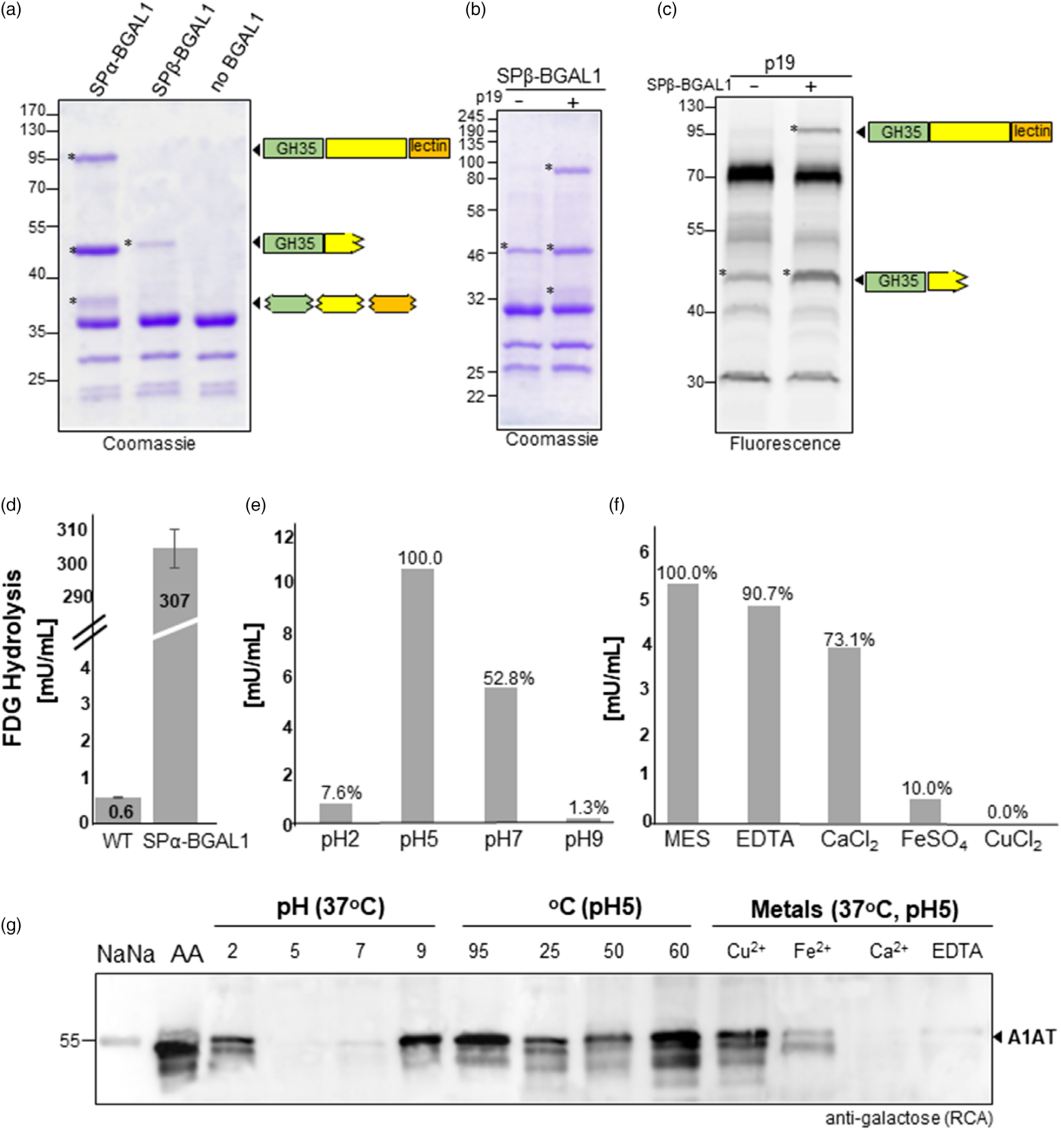
Characterization of *Nb*BGAL1 β‐galactosidase activity. (a) Coomassie staining of secreted proteins (AF) in *Nicotiana benthamiana* plants expressing SPα‐BGAL1, SPβ‐BGAL1 and empty vector. Protein bands identified as BGAL1 by peptide mapping are marked (*). Bands smaller than 40 kDa are associated with *agrobacteria* infection. Peptides originated from different domains of BGAL1 identified and mapped by mass spectrometry are shown on the right. (b) Coomassie staining of AF from *N. benthamiana* plants expressing SPβ‐BGAL1 without (−) and with (+) co‐expression of p19. (c) Glycosidase activity profiling of AF from *N. benthamiana* plants expressing (1) p19 and (2) co‐expressing p19 with SPβ‐BGAL1. Protein bands identified as BGAL1 by peptide mapping are marked (*). Protein size marker is shown in kilo Dalton (kDa). (d) β‐Galactosidase activity was measured in AF isolated from leaves of *N. benthamiana* wild‐type (WT) and from *N. benthamiana* transiently expressing SPα‐BGAL1 by FDG assay. Error bars represent SEM of n = 3 biological replicates. (e) FDG assay was used to measure the optima pH for SPα‐BGAL1 activity. Maximum activity at pH 5.0 was set to 100% to calculate the relative activity at other pHs. (f) The effect of various metal ions on the activity of AF‐derived SPα‐BGAL1 was studied by FDG in the presence of metal ions. Maximum activity obtained with no supplement (MES) was set to 100% to calculate relative activity in the presence of divalent metal ions (Fe^2+^ Ca^2+^, Cu^2+^) and EDTA. A β‐Galactosidase purified from *Aspergillus oryzae* (276 U/mL) was used as a standard to quantify SPα‐BGAL1 activity in mU/mL of AF. (g) The effects of different pHs, temperatures and metals on the β‐galactosidase activity of SPα‐BGAL1 were assayed with a galactose‐binding lectin (RCA). Sialylated human A1AT (NaNa, negative control) was digested with neuraminidase to expose galactose residues (AA, positive control). The levels of galactosylation on A1AT *N‐*glycans were compared before and after incubating the asialo‐A1AT (AA) with SPα‐BGAL1 in different conditions (see also Figure S6).

Full‐length *Nb*BGAL1 (25‐846 amino acids) and the *Nb*BGAL1‐GH35 domain (25‐360 amino acids) were expressed in *N. benthamiana* using tobacco mosaic virus (TMV)‐based magnICON®‐assembled vectors carrying either the β‐ or α‐signal peptide (SPβ‐BGAL1, SPα‐BGAL1 and SPα‐BGAL1‐GH35; Figure S1). Coomassie staining of secreted proteins isolated from AF and subsequent peptide mapping demonstrates that SPα‐BGAL1 accumulates as three protein bands: a 95‐kDa corresponding to the full‐length protein sequence; a 48‐kDa band assigned to the GH35 domain lacking its *C*‐terminus and a 38‐kDa protein band representing a mixture of *N*‐ and *C*‐ protein truncations (Figure 2a). In contrast, SPβ‐BGAL1 expressed is detected as a single 48‐kDa protein and all the identified peptides were originated from the GH35 catalytic domain and not from the *C*‐terminal half of this protein (Figure 2a). To evaluate whether this discrepancy is due to differences in the expression level between SPα‐BGAL1 and SPβ‐BGAL1, we co‐expressed the p19 RNA silencing suppressor protein, known to increase levels of transient expression (Voinnet *et al.*, 2003). Indeed, p19 significantly boosted SPβ‐BGAL1 protein accumulation, which is now detected as a 95‐kDa full‐length protein in addition to the truncated 48‐kDa protein (Figure 2b). Notably, despite several attempts to express the GH35 catalytic domain of *Nb*BGAL1 (SPα‐BGAL1‐GH35) no protein was detected in AF or in TSP (Figure S2).

### Characterization of *Nb*BGAL1 β‐galactosidase activity

Many enzymes involved in protein glycosylation are themselves glycoproteins. Using the NetOGlyc 4.0 Server (www.cbs.dtu.dk/services/NetNGlyc), we predicted four potential glycosylation sites on *Nb*BGAL1, located at Asn^26^, Asn^255^, Asn^580^ and Asn^723^ (Figure S3a). We therefore evaluated the glycosylation profile of *Nb*BGAL1 expressed in ΔXTFT and ΔXTFT^GAL^ plants. Trypsin digestion of full‐length SPα‐BGAL1 isolated from AF (95‐kDa, Figure 2a) allowed the identification of three out of the four glycopeptides (GP1, 3 and 4; Table S1). Glycoprofiling of SPα‐BGAL1 by LC‐ESI‐MS showed that all GPs are decorated with complex glycans with one or two terminal GlcNAc residues. Interestingly, SPα‐BGAL1 expressed in ΔXTFT^GAL^ plants lacks terminal β1,4‐galactose residues in all GPs (Figure S3b).

Next, *Nb*BGAL1 was evaluated for β‐galactosidase activity using a fluorogenic substrate for β‐galactosidases. We confirmed that SPα‐BGAL1 has β‐galactosidase activity since it can cleave galactose from fluorescein di‐β‐D‐galactopyranoside (FDG) when overexpressed in apoplast of *N. benthamiana* (Figure 2d)*.* Approximately 0.6 mU of endogenous *Nb*BGAL1 (WT) is naturally active in 1 mL of AF, and this is markedly increased to up to 300 mU/mL upon overexpression of SPα‐BGAL1 (Figure 2d).

Studies of the enzyme kinetics determined the apparent K_M_ and V_max_ values of AF‐derived SPα‐BGAL1 for FDG as 29.8 mm and 2.7 µm/min/mL of AF, respectively.

β‐Galactosidase activity assayed by FDG hydrolysis at various pH shows that SPα‐BGAL1 optimal hydrolysis is obtained at pH5.0. The FDG hydrolytic activity was halved at near‐neutral pH and markedly reduced at extreme acidic pH (7.6% at pH 2.0), and only 1.5 % of full enzyme activity was observed at pH 9.0 (Figure 2e).

The effects of various metal ions and EDTA on enzyme activity were studied by incubating AF from leaves overexpressing SPα‐BGAL1 with FDG in the presence of 5.0 mm metal ions. EDTA has no significant effect on the hydrolytic activity; Mg^2+^ and Ca^2+^ slightly reduce SPα‐BGAL1 activity (30%), while Fe^2+^ and Cu^2+^ significantly inhibited SPα‐BGAL1 activity (90% inhibition for Fe^2+^ and no activity detected in the presence of Cu^2+^) (Figure 2f). We confirmed the optimal pH and the impact of metal ions on SPα‐BGAL1 activity by the ability of the enzyme to remove β1,4‐galactose residues present on plasma‐derived alpha‐1 anti‐trypsin (A1AT) after removing terminal sialic acids by sialidase treatment (Figure 2g). Incubation of galactosylated A1AT (AA: Gal_2_GlcNAc_2_Man_3_GlcNAc_2_) with AF‐derived SPα‐BGAL1 at different temperatures shows that the SPα‐BGAL1 optimum activity is at 37^ᵒ^C. SPα‐BGAL1 enzymatic activity is reduced at lower temperature (25 ^ᵒ^C); lower at relatively increased temperatures (up to 50^ᵒ^C); and inhibited at higher temperatures (from 60 ^ᵒ^C and above) (Figure 2g).

The *in vitro* β‐galactosidase activity assay described above does not discriminate if both forms of SPα‐BGAL1 (full‐length and GH35 domain) protein are active. The active state of glycosidases isolated from apoplast has been previously monitored, indicating that the truncated 48‐kDa *Nb*BGAL1 is an active β‐galactosidase (Buscaill *et al.*, 2019; Chandrasekar *et al.*, 2014). It is thought that that trimming of the *C*‐terminal half of BGAL generates a mature and active protein consisting only of the GH35 catalytic domain.

Here, we aimed to investigate whether the full‐length version of the protein, carrying the C‐terminal domain, is active. However, our attempts to use a *C*‐terminal Strep‐tag to purify the full‐length SPα‐BGAL1 protein were unsuccessful. To overcome this shortcoming, we used glycosidase activity profiling with a fluorescent glycosidase probe (Chandrasekar et al., 2016) to specifically identify active β‐galactosidases in AFs expressing p19 or co‐expressing p19 and SPβ‐BGAL1. Both AF samples showed a 45‐kDa fluorescent protein signal corresponding to the GH35 domain of *Nb*BGAL1, but we detected an additional signal at 95 kDa upon overexpression of SPβ‐BGAL1 (Figure 2c). These data demonstrate that full‐length *Nb*BGAL1 has β‐galactosidase activity.

### Low levels of human‐like β1,4‐galactosylation in planta

Full monoclonal antibodies co‐expressed with ^ST^GalT or expressed in ^ST^GalT transgenic plants are up to 83% galactosylated from which 60% are di‐galactosylated (AA) (Schneider *et al.*, 2015). These results contrast strongly with the low galactosylation levels of other glycoproteins in *N. benthamiana*.

To illustrate this, we expressed five different recombinant glycoproteins in ∆XTFT plants stably transformed with a modified version of the human β1,4‐galactosyltransferase. These recombinant plants are similar to those described previously (^ST^GalT‐ΔXF) (Schneider *et al.*, 2015), but in this new host plant, the catalytic domain of the GalT was targeted to the late Golgi using the cytoplasmic tail, transmembrane domain and stem (CTS) region of the Arabidopsis β1,3‐galactosyltransferase (GALT1). ∆XTFT plants stably transformed with ^GALT1^GalT (∆XTFT^GAL^) behave very similar to ^ST^GalT‐ΔXF with no major differences in growth or developmental phenotypes (Schneider *et al.*, 2015).

**Figure 3:**
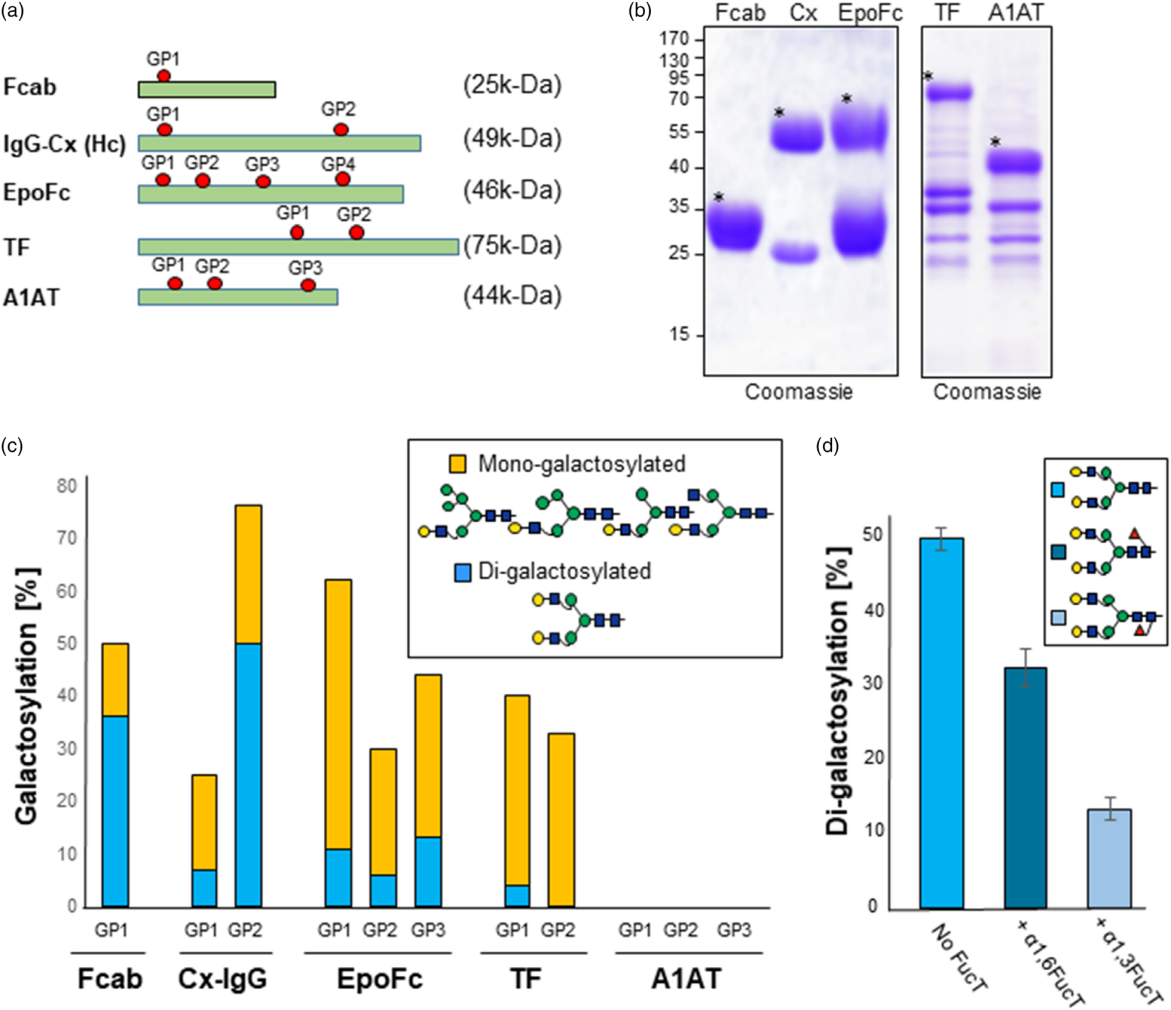
Poor β1,4‐galactosylation of reporter glycoproteins expressed in ΔXTFT^GAL^. (a) Schematic representation of reporter glycoproteins used in this study. Glycosylation sites and theoretical molecular weight of mature unglycosylated proteins are indicated (see also Table S1). (b) Coomassie staining of Fcab‐Her2, Cx‐IgG and EpoFc purified by protein A immunoaffinity out of total soluble proteins and of TF and A1AT isolated by collecting the apoplastic fluid of infiltrated leaves. Protein bands used for glycan analysis are marked (*). (**c**) Relative abundance (%) of β1,4‐galactosylation (mono‐ and di‐antennary) on reporter proteins transiently expressed in ∆XTFT^GAL^ transgenic plants. (d) Relative abundance (%) of di‐galactosylation of Cx‐IgG‐Fc expressed in the presence or absence of core fucose. Cx‐IgG was transiently co‐expressed without or with plant‐(α1,3‐FucT) and mammalian‐(α1,6‐FucT) core fucosyltransferases. For detailed information, see Figures S4 and S5. For interpretation of glycoforms present in assigned peaks, see Figure S13.

We next used viral‐based binary vectors (magnICON®) to transiently express five different recombinant proteins that carry a different number of glycosylation sites: i) human transferrin (TF); ii) erythropoietin fused to an Fc fragment (EpoFc); iii) human alpha‐1 anti‐trypsin (A1AT); iv) monoclonal antibody cetuximab (Cx‐IgG); and v) the Fc fragment with engineered HER2/neu‐binding sites (Fcab‐HER2 or in short Fcab) (Figure 3a and Table S1, Castilho *et al.*, 2011b, 2013, 2014, 2015; Jez *et al.*, 2012). The recombinant proteins were transiently expressed in ∆XTFT^GAL^ plants and either purified from TSP (Fcab‐Her2, EpoFc, Cx‐IgG) or collected in AF (TF and A1AT) (Figure 3b). Liquid chromatography–electrospray ionization mass spectrometry (LC‐ESI‐MS) revealed that only 8% of *N‐*glycans are di‐galactosylated for the Cx‐IgG glycosite located on the variable fraction (Fab) of the heavy chain (GP1) (Figure S4 and Figure 3c). By contrast, the GP2 site of Cx‐IgG shows up to 50% of di‐antennary galactosylated (AA) structures. Galactosylation of the Fc glycosite on Fcab‐Her2 is also not efficient as only 36% of the *N‐*glycans are fully di‐galactosylated. Similarly, galactosylation of the three glycosites of the Epo fragment on EpoFc is very inefficient and only 12% of the sites are di‐galactosylated. Although TF can be galactosylated with mono‐antennary hybrid structures, di‐galactosylation was very poor in TF and not detected in A1AT. All together, these data indicate that the efficiency of galactosylation is poor and varies from protein to protein, and even between different glycosites of a particular protein.

### Core fucosylation suppresses galactosylation

Most mammalian glycoproteins carry complex *N*‐glycans decorated with a core α1,6‐fucose residue, while in plant proteins, glycans can be decorated with a α1,3‐fucose residue. We previously demonstrated that the α1,3‐fucose residue promotes sialylation in ΔXTFT plants that express the human sialylation pathway (Castilho *et al.*, 2015). Galactosylation is the intermediate step necessary for protein sialylation. To determine whether fucosylation also affects galactosylation, Cx‐IgG was transiently expressed in ∆XTFT^GAL^ plants in the absence of core fucose or co‐expressed with α1,3‐FucT and α1,6‐FucT fucose transferases. Glycoprofiling showed that core fucosylation negatively influences Fc‐galactosylation: The level of bi‐antennary galactosylated glycans is reduced upon co‐expression with α1,6‐FucT (27%, AAF^6^) or α1,3‐FucT (12% AAF^3^) when compared to 50% di‐galactosylated Fc‐glycans in the absence of fucosyltransferases (Figure 3d and Figure S5). These data indicate that the core fucosylation negatively affects the maintenance of galactosylated glycans.

### *Nb*BGAL1 removes terminal β1,4‐galactose residues on recombinant *N‐*glycoproteins

**Figure 4:**
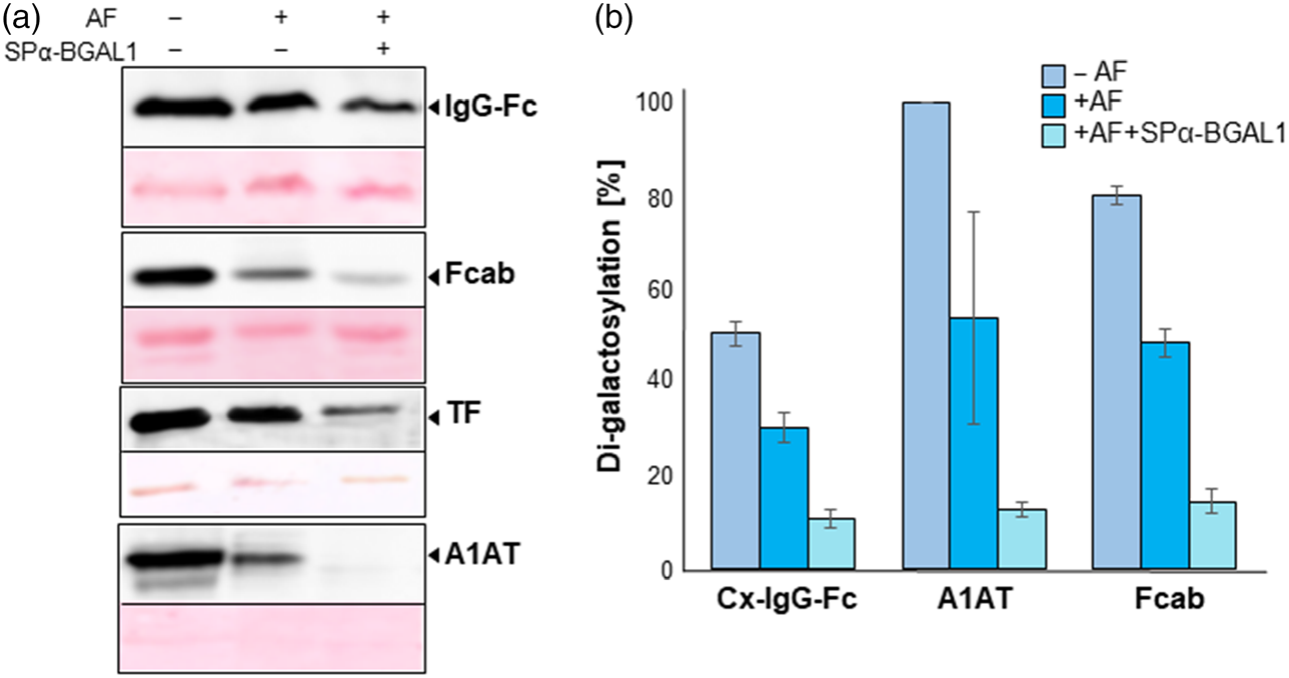
*Nb*BGAL1 removes terminal β1,4‐galactose residues on recombinant *N‐*glycoproteins *in vitro*. (a) RCA lectin blotting was used to assess galactosylation levels in different reporter proteins. Galactosylated IgG, Fcab, TF and A1AT (‐AF) were incubated with AF collected from WT *Nicotiana benthamiana* (+AF) or with AF collected from WT *N. benthamiana* expressing SPα‐BGAL1 (AF + SPα‐BGAL1). Ponceau staining shows similar amounts of protein loaded. (b) Relative abundance (%) of β1,4‐galactosylated *N*‐glycans on selected reporter proteins. *In vitr*o activity of endogenous and recombinant *Nb*BGAL1 was assessed by determining the levels of di‐galactosylated N‐glycans (AA) on reporter proteins before (‐AF) and after *in vitro* incubations with endogenous level of *Nb*BGAL1 from plant apoplast (+AF) or overexpressed in *N. benthamiana* plants (+AF + SPα‐BGAL1). Di‐galactosylated A1AT and Fcab were obtained by digestion of the sialylated versions with neuraminidase. Values for A1AT are an average of galactosylation on the three glycosites (GP1‐3). Detailed mass spectrometry for Fcab‐Her2 and A1AT is shown in Figure S6. For interpretation of glycoforms present in assigned peaks, see Figure S13.

As shown above, the galactosylation levels of plant‐derived IgG‐Fab, EpoFc, Fcab‐Her2, TF and A1AT differ considerably from the high levels observed on IgG‐Fc. To address the question whether galactosylated *N*‐glycans are substrates for *Nb*BGAL1 and evaluate its ability to remove terminal β1,4‐galactose residues *in vitro,* we incubated galactosylated proteins with AF from WT *N. benthamiana* and from *N. benthamiana* overexpressing SPα‐BGAL1 and compared their galactosylation levels using a galactose‐binding lectin *Ricinus communis* agglutinin (RCA)**.** The results for IgG‐Fc showed lower levels of galactosylation upon incubation with recombinant SPα‐BGAL1, but the relative levels of galactosylation do not differ significantly after incubation with endogenous BGAL activity present in the AF (Figure 4a). To assess the activity of *Nb*BGAL1 also on Fcab‐Her2, TF and A1AT, we used sialylated versions of the proteins and removed sialic acid by sialidase digestion to expose terminal β1,4‐galactose residues (Figure S6). When compared to IgG‐Fc, these proteins are more susceptible to the activity of *Nb*BGAL1 (Figure 4a). Protein glycosylation profiles reveal a significant reduction of di‐galactosylated glycans for all glycopeptides due to the activity of native (AF) and overexpressed *Nb*BGAL1 (AF + SPα‐BGAL1) (Figure 4b, Figure S6 and Table S2), demonstrating that galactosylated *N*‐glycans are substrates for *Nb*BGAL1.

**Figure 5:**
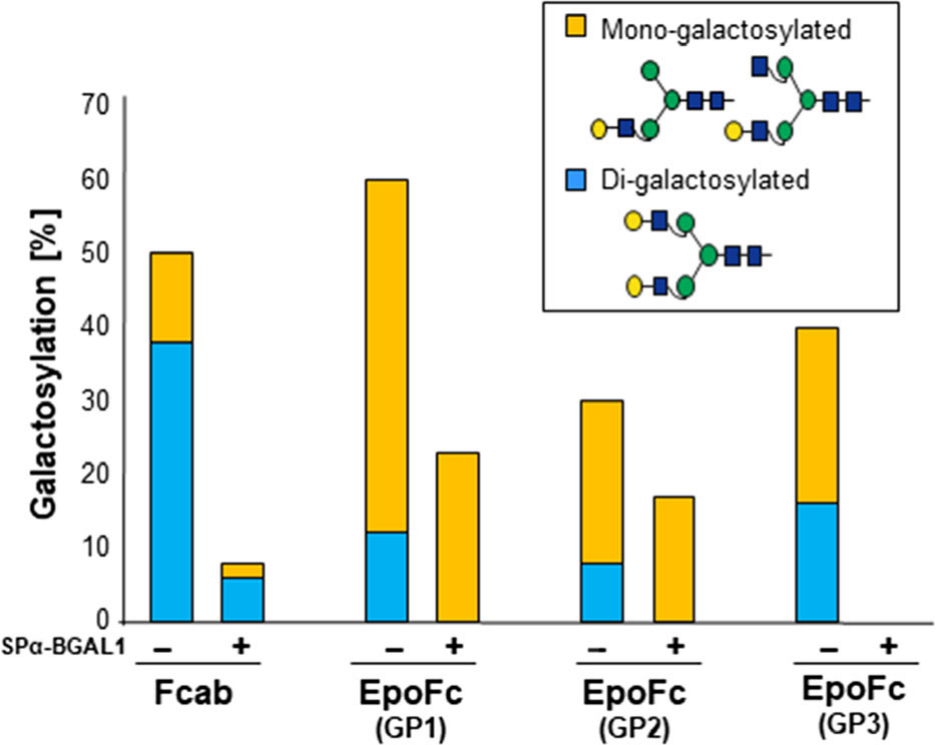
*Nb*BGAL1 removes terminal β1,4‐galactose residues on recombinant *N‐*glycoproteins *in planta*. Relative abundance (%) of β1,4‐galactosylated (mono‐ and di‐antennary) *N*‐glycans on Fcab and EpoFc co‐expressed with (+) or without (−) SPα‐BGAL1 in ∆XTFT^GAL^ transgenic plants. For detailed glycoprofiles, see Figure S7. For interpretation of glycoforms present in assigned peaks, see Figure S13.

Next, to evaluate whether recombinant *Nb*BGAL1 is active *in vivo,* we co‐expressed reporter glycoproteins with or without SPα‐BGAL1 in ΔXTFT^GAL^ plants and compared the β1,4‐galactosylation levels by LC‐ESI‐MS. To avoid expression of competitive virus, reporter glycoproteins were co‐expressed with SPα‐BGAL1 cloned into non‐viral‐based binary vectors (Figure S1). Overall, a drastic reduction of mono‐ and di‐galactosylated glycans was observed upon co‐expression of SPα‐BGAL1 or SPβ‐BGAL1 (Figure 5 and Figure S7), demonstrating that *Nb*BGAL1 is active *in vivo*. Moreover, analysis of secreted endogenous proteins present in AF from ∆XTFT^GAL^ plants showed that galactosylation levels of 18% are further reduced to 6% upon expression of SPα‐BGAL1 (Figure S8). All together, these data demonstrate that *Nb*BGAL1 can remove terminal β1,4‐galactose residues on recombinant *N*‐glycoproteins.

### *Nb*BGAL1 removes terminal β1,3‐galactose residues from Lewis a epitopes on *N‐*glycans and from mucin‐type *O*‐glycans

In contrast to the occurrence of β1,4‐galactosyl residues in animal complex‐type *N‐*glycans, plant‐derived glycoproteins, especially secreted‐type glycoproteins, may carry a β1,3‐galactosyl residue as part of the so‐called Lewis a (Le‐a) epitopes. These are terminal trisaccharides consisting of α1,4‐fucose and β1,3‐galactose linked to *N‐*acetylglucosamine (Galβ1‐3(Fucα1‐4)GlcNAcβ1‐) (Fitchette‐Lainé *et al.*, 1997; Strasser *et al.*, 2007a), a carbohydrate formation previously detected on EpoFc (Castilho *et al.*, 2011b).

Here, we have expressed EpoFc in WT *N. benthamiana* and detected significant amounts of structures compatible to Le‐a by immunoreaction to JIM84.

The β1,3‐galactosyltransferase gene responsible for the biosynthesis of the Lewis a in Arabidopsis has been identified (Strasser *et al.*, 2007a), but the β‐galactosidase responsible for the degradation of *N‐*glycans harbouring the Le‐a epitope remains to be identified.

**Figure 6:**
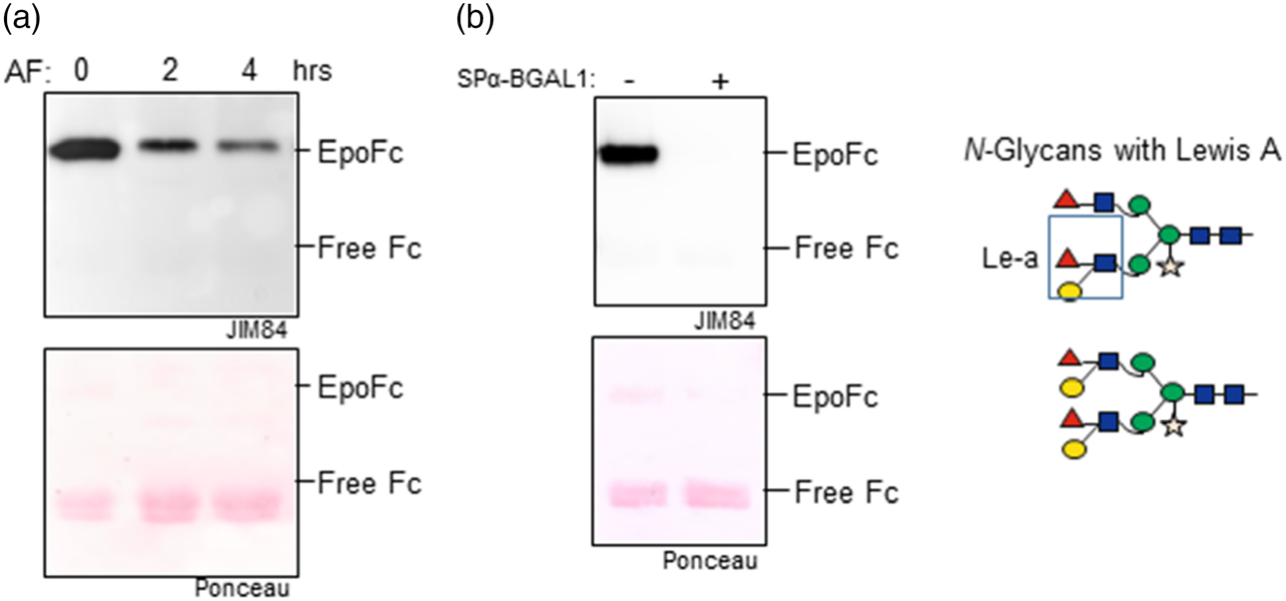
*Nb*BGAL1 removes terminal β1,3‐galactose residues from Lewis a motifs. (a) EpoFc was expressed in *Nicotiana benthamiana* WT plants, and the synthesis of Lewis a motifs was monitored by glycan analysis*.* Purified EpoFc carrying *N*‐glycans with Lewis a motifs was incubated with AF isolated from *N. benthamiana* WT plants for 2 or 4 h. The relative amounts of Lewis a motifs were monitored by Western blotting with anti‐Lewis a antibodies (JIM84) (b) EpoFc was co‐expressed in *N. benthamiana* without (−) and with (+) SPα‐BGAL1. Ponceau staining show similar amount of proteins loaded. Bands corresponding to full‐length fusion protein (EpoFc) and degradation product (free Fc) are marked. Cartoons representing *N*‐glycans carrying Lewis a motifs are shown on the right. Mass spectrometry showing the relative levels of Lewis a epitopes for Epo GP3 is shown in Figure S9. For interpretation of glycoforms present in assigned peaks, see Figure S13.

To investigate whether *Nb*BGAL1 can remove the β1,3‐galactose residues present in the EpoFc Le‐a, we incubated the purified EpoFc protein with AF isolated from WT *N. benthamiana* and monitored the amounts of Le‐a using the anti‐Le‐a antibody JIM84. A decrease in the Le‐a signal was immediately detected after two hours of incubation, which became more evident after 4 hours (Figure 6a), indicating that *Nb*BGAL1 can remove the β1,3‐galactose residues present in the EpoFc Le‐a. Next, to evaluate the activity of *Nb*BGAL1 *in planta*, EpoFc was transiently co‐expressed in *N. benthamiana* with and without SPα‐BGAL1. The 55‐kDa band corresponding to the intact EpoFc is detected using JIM84 only when SPα‐BGAL1 is not overexpressed (Figure 6b), indicating that the synthesis of Le‐a in EpoFc is prevented or reduced by SPα‐BGAL1 activity. These results were confirmed by LC‐ESI‐MS where glycan profiles showed that Le‐a epitopes are not detected on EpoFc upon co‐expression with SPα‐BGAL1 (Figure S9). All together, these data demonstrate that *Nb*BGAL1 removes terminal β1,3‐galactose residues from Lewis a epitopes on *N*‐glycans.

*O*‐glycosylation is a common post‐translational modification of serine and threonine residues of secreted and membrane‐bound proteins (Strasser, 2012; Strasser, 2013). Many glycoproteins carry glycans initiated by GalNAc attached to the hydroxyl of Ser or Thr residues, also called *O*‐glycans.

Although plants do not contain endogenous glycosyltransferases that perform mammalian‐type Ser/Thr glycosylation, the synthesis of plant‐derived EpoFc carrying mucin‐type *O*‐glycan terminated by galactose (core 1 or T‐antigen) was previously described (Castilho *et al.*, 2012).

To investigate whether *Nb*BGAL1 is able to act on EpoFc *O*‐glycans to remove the terminal β1,3‐galactose residue present on T‐antigen glycans, we transiently co‐expressed EpoFc with a mammalian GalNAc‐transferase (GalNAc‐T2) and a drosophila core 1 β1,3‐galactosyltransferase (C1GALT1) (Castilho *et al.*, 2012). After protein A purification and confirmation of the synthesis of T‐antigen (GalNAc + Gal) by mass spectrometry, EpoFc was incubated at 37ᵒC with AF from *N. benthamiana* plants expressing SPα‐BGAL1. Glycan analysis revealed that *Nb*BGAL1 is able to reduce the T‐antigen present in EpoFc from 60% to 40% in 1 h and to 14% in 4 h (Figure S10), demonstrating that *Nb*BGAL1 can remove terminal β1,3‐galactose residues from mucin‐type *O*‐glycans.

### Suppression of *Nb*BGAL1 activity increases the abundance of β1,4‐galactosylated *N*‐glycans

**Figure 7:**
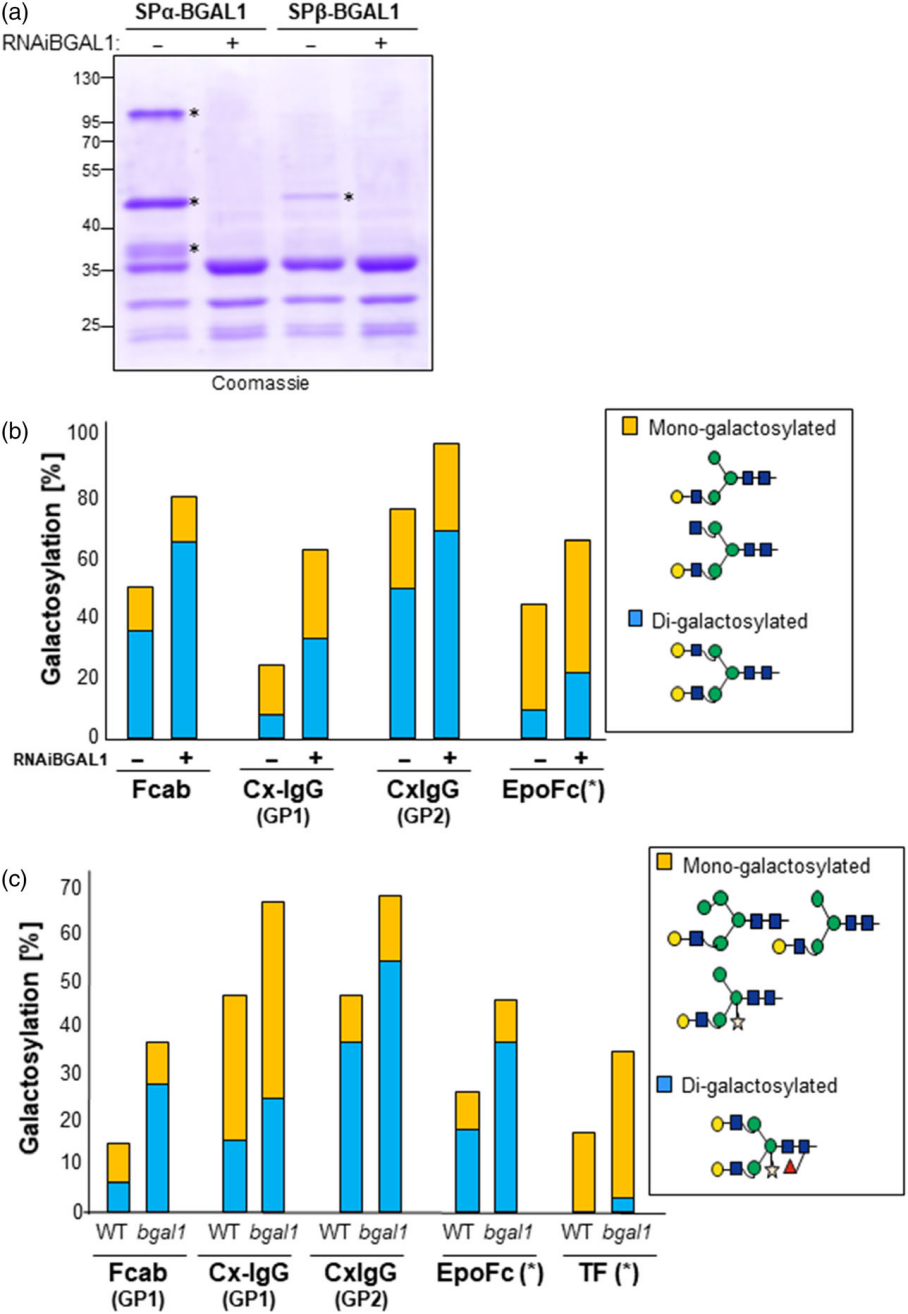
Depletion of *Nb*BGAL1 expression increases the abundance of β1,4‐galactosylated *N*‐glycans. (a) *In planta* down‐regulation of BGAL1 expression. Coomassie staining of secreted proteins from *Nicotiana benthamiana* leaves infiltrated with SPα‐BGAL1 and SPβ‐BGAL1 without (−) or with (+) RNAiBGAL1. Protein bands identified as BGAL1 by peptide mapping are marked (*). Protein size marker (M) is shown in kilo Dalton (kDa). (b) Relative abundance (%) of β1,4‐galactosylation (mono‐ and di‐antennary) on selected reporter proteins after suppression of NbBGAL1 expression by RNA interference: Fcab, Cx‐IgG and EpoFc were co‐expressed with (+) or without (−) RNAiBGAL1 in ∆XTFT^GAL^ transgenic plants. (*) Values for EpoFc were calculated as an average of galactosylation on the three glycosites (GP1‐3). (c) Relative abundance (%) of β1,4‐galactosylation (mono‐ and di‐antennary) on selected reporter proteins co‐expressed with ^ST^GalT in *N. benthamiana* WT and BGAL1 null mutant (*bgal1*) plants. (*) Values for EpoFc and TF were calculated as an average of galactosylation on all glycosites. Detailed mass spectrometry is shown in Figures S11 and S12. For interpretation of glycoforms present in assigned peaks, see Figure S13.

To investigate whether the formation of β1,4‐galactosylated *N*‐glycans can be improved by silencing *Nb*BGAL1, a hairpin construct for *Nb*BGAL1 (RNAiBGAL1; Figure S1) was generated. Transient RNAiBGAL1 expression drastically reduces the accumulation of transiently expressed BGAL1 (Figure 7a), demonstrating that the RNAiBGAL1 construct is effective.

Co‐expression of RNAiBGAL1 with the five mammalian reporter proteins increased the level of mono‐ and di‐galactosylation in these proteins, especially for glycosites that are normally poorly galactosylated (Figure 7b and Figure S11). For instance, we detect an increase from 8% to 33% in the Cx‐Fab glycosite.

To investigate whether galactosylation of recombinant proteins is also increased in the *bgal1* null mutant plants (*bgal1‐1*), we compared the galactosylation level of reporter glycoproteins transiently co‐expressed in WT and in *bgal1‐1*. Since both these host plant do not express the β1,4‐galactosyltransferase, we co‐expressed the recombinant proteins with ^ST^GalT, driven by a weak promoter to promote enhanced galactosylation (Act:: ^ST^GalT; Kallolimath *et al.*, 2018). When expressed in *bgal1‐1* mutant plants, the levels of di‐galactosylation are significantly improved for all reporter glycoproteins: EpoFc (from 18% to 37%); TF (from 0% to 12%); Cx‐IgG (from 37% to 56% in Fc and 15% to 25% in Fab); and Fcab‐HER2 (from 6% to 28%) (Figure 7c and Figure S12). Importantly, these levels of galactosylation also depend on the transient expression of ^ST^GalT. In agroinfiltration experiments, we cannot assure that all cells are simultaneously expressing ^ST^GalT and the reporter protein. In fact, we observed a significant discrepancy on the di‐galactosylation levels in independent experiments. Nonetheless, our combined results using RNAiBGAL1 and *bgal1‐1* null mutant plants clearly demonstrate that *N. benthamiana Nb*BGAL1 activity is a critical factor for the trimming of terminal β1,4‐galactose residues on *N‐*glycans of recombinant glycoproteins in plants.

## Discussion

*Nicotiana benthamiana* is currently the favourite and most widely used plant host when it comes to the production of recombinant proteins with engineered glycosylation. Tailored glycosylation profiles on *N. benthamiana* ‐derived therapeutic proteins have been reported including the extensive engineering of *in planta* multi‐antennary sialylation (Castilho *et al.*, 2013; Castilho *et al.*, 2014; Schneider *et al.*, 2013) and polysialylation (Kallolimath *et al.*, 2016). However, except for monoclonal antibodies, none of these studies reported successful human‐like β1,4‐galactosylation.

Based on recent findings on the profiling of active glycosidases in the apoplast of *N. benthamiana* (Buscaill *et al.*, 2019; Chandrasekar *et al.*, 2014), we set up to evaluate and characterize *Nb*BGAL1 as one of the potential enzymes involved in processing galactosylated glycans.

We used viral‐based vectors to express *Nb*BGAL1 (SPβ‐BGAL1 or SPα‐BGAL1). Studies have shown that signal peptides can profoundly impact protein secretion (Kober *et al.*, 2013). Thus, the efficiency of protein secretion can be improved by choosing an optimized signal peptide (Haryadi *et al.*, 2015; Klatt and Konthur, 2012). This was observed for the overexpression of BGAL1 in *N. benthamiana*, where high level of expression of full‐length BGAL1 was only detected for the chimeric protein (SPα‐BGAL1). However, the expression level of SPβ‐BGAL1 was efficiently improved by co‐expression with silencing inhibitor p19.

In this study, we show that *Nb*BGAL1 is a secreted and active beta‐galactosidase acting on both *N*‐ and *O*‐glycans. *Nb*BGAL1 removes terminal β1,4‐galactose residues on recombinant *N*‐glycoproteins *in vitro* and *in planta.* In addition, *Nb*BGAL1 removes terminal β1,3‐galactose residues from Lewis a epitopes on *N‐*glycans and from mucin‐type *O*‐glycans.

Our attempts to determine whether the catalytic domain of BGAL1 (GH35) is sufficient for trimming terminal galactose residues were unsuccessful since expression of the *Nb*BGAL1 GH35 domain lacking the *C*‐terminus could not be achieved. Studies of the processing of a human lysosomal BGAL expressed in COS‐1 cells suggested that the proteolytic processing of the full‐length protein to remove the *C*‐terminus is essential for activity (van der Spoel *et al.*, 2000). Also here, expression of the human BGAL *N‐*terminal catalytic domain without the *C*‐terminal domain could not be achieved in COS‐1 cells (van der Spoel *et al.*, 2000). In contrast, transfection of COS‐1 cells with DNA fragments representing the *N‐* and *C*‐terminal domains increased the overall β‐galactosidase activity, implying that the catalytic activity of BGAL requires an interaction of the two domains.

Characterization of *Nb*BGAL1 showed that the enzyme is a secreted glycoprotein with four potential glycosylation sites decorated with complex‐type *N*‐glycans. Interestingly, no galactosylation was detected on *Nb*BGAL1 when expressed in ∆XTFT^GAL^ plants, which could be due to the enzyme activity on its own glycans. The optimal *in vitro Nb*BGAL1 activity was established at acidic pH (5.0), which corresponds to the leaf apoplastic pH (Grignon and Sentenac, 1991).

A common obstacle that hampers detailed research on the impact of glycosylation to protein functional activities is the incomplete knowledge of how glycan structures are generated and the unavailability of expression platforms that allow the synthesis of targeted glycoforms.

Studies on the impact of galactosylated *N*‐ or *O*‐glycans on protein function are few since terminal galactose residues are either capped by sialic acid or trimmed off by galactosidases.

Examples are studies comparing the performance of asialo‐ and sialylated EpoFc (Castilho *et al.*, 2013), BChE (Schneider *et al.*, 2013), IgM (Loos *et al.*, 2014) and A1AT (Castilho *et al.*, 2014) that do not include the galactosylated (intermediate) glycoform. Another investigation comparing the synthesis core‐fucosylated asialo‐ and sialylated Cx‐IgG again excluded the fucosylated–galactosylated counterpart (Castilho *et al.*, 2015). The efficiency of glycoengineering depends on the protein and on the glycosylation site. A dramatic example was our efforts to β1,4‐galactosylate A1AT (Castilho *et al.*, 2014). Recombinant A1AT expressed in ΔXTFT and in ΔXTFT^GAL^ is decorated mainly with paucimannosidic *N‐*glycans contrasting with the highly efficient sialylation in ΔXTFT^SIA^ (Castilho *et al.*, 2014). This indicates that capping β1,4‐galactose residues with sialic acid prevents their exposure to galactosidases and consequently the accessibility of hexosaminidases to GlcNAc residues. Another example is EpoFc: the poor galactosylation observed for three glycosites located on the Epo fragment contrasts with efficient galactosylation on the Fc site. By contrast, EpoFc expressed in ΔXTFT^SIA^ shows fully sialylated Epo glycosites, while Fc fragment is decorated mainly with mono‐sialylated glycans (Castilho *et al.*, 2013). Even for monoclonal antibodies, glycoengineering greatly depends on the availability of the glycosite for glyco‐modulating enzymes (Castilho *et al.*, 2015). While glycans connected to the IgG‐Fab fragment of Cx‐IgG are exposed to the surrounding solvent, the structures located in the Fc fragment are largely shielded by the opposing CH2 fragment, and therefore, galactosylation at this site is much more efficient. Interestingly, the exposure of Fc to glyco‐modelling enzymes seems to depend on core fucosylation. Here, we showed that Fc *N‐*glycans can also be converted into BGALs substrates upon plant‐specific core α1,3‐fucosylation. We have previously shown that *N‐*linked glycans on IgG opposite chains (CH2) maintain the conformation of the Fc domain and impose constraints on the action of processing enzymes, thereby hampering synthesis of more complex glycans (Castilho *et al.*, 2015). However, plant‐specific core fucosylation seems to alleviate these structural constraints making terminal sugar residues available not just to glycosyltransferases (e.g. GnT*‐*III, IV V and ST) (Castilho *et al.*, 2015) but also to glycosidases (e.g. HEXO3; Shin *et al.*, 2017).

In conclusion, active BGALs are a severe limitation for tailored glycosylation because these enzymes generate truncated glycans and compromise the synthesis of galactosylated *N*‐ and *O*‐glycans. We showed that co‐expression of the RNAiBGAL1 construct resulted in increased levels of galactosylated *N*‐glycans for all tested glycoproteins and glycosites. Moreover, when expressed in *bgal1* mutant plants with a WT background, galactosylation of recombinant proteins is greatly increased. The impact of terminal galactosylation on the function of recombinant proteins has only been determined for IgG1 (Thomann *et al.*, 2016). The results presented here are highly encouraging for a future establishment of yet another ΔXTFT‐based expression platform, depleted of β‐galactosidase activity, thus enabling β1,4‐galactosylation on a diverse group of glycoproteins. This study opens a new area for plant‐based glycoengineering.

## Material and methods

### Plant material

In this investigation, we used *Nicotiana benthamiana* wild‐type plants (WT) and mutant plants lacking plant‐specific core β1,2‐xylose and α1,3‐fucose residues (ΔXTFT; Strasser *et al.*, 2008). For *in planta* β1,4‐galactosylation, the cytoplasmic tail, transmembrane domain and stem (CTS) region of the rat α2,6‐sialyltransferase present in the ^ST^GalT construct (Strasser *et al.*, 2009) was replaced by the CTS region (amino acids 1‐60) of the Arabidopsis α1,3‐galactosyltransferase (GALT1). GalT1 F1/ GalT1 R1 primer pair (Table S3) was used to amplify the CTS region, and the resulting ^GALT^GalT fragment was recloned in binary vector carrying an expression cassette for glyphosate tolerance (Dr. Koen Wetering, Bayer CropScience). Finally, ΔXTFT plants were stably transformed with ^GALT^GalT construct to generate the ΔXTFT^GAL^ expression platform.

Null mutants of *N. benthamiana* plants lacking β‐galactosidase 1 activity (*bgal1‐1*) were generated using genome editing with CRISPR/Cas9 as described recently (Buscaill *et al.*, 2019). Four‐ to 5‐week‐old plants were used for agroinfiltration experiments.

### Cloning of reporter glycoproteins used in this investigation

We expressed five different reporter recombinant glycoproteins in *N. benthamiana*. (TMV)‐MagnICON®‐assembled viral vector system was used to express TF (Castilho *et al.*, 2011b), EpoFc (Castilho *et al.*, 2013) and A1AT (Castilho *et al.*, 2014). Two non‐competitive virus vectors, TMV‐ and potato virus X (PVX)‐based, were used to express the heavy and light chains of Cx‐IgG (Castilho *et al.*, 2015). In addition, we recloned an Fc fragment with engineered HER2/neu‐binding sites (Fcab‐HER2; Jez *et al.*, 2012) into the PVX‐based magnICON®‐assembled vector. For that, we amplified the cDNA sequence, with flanking *Bsa*I restriction sites, out of the previous vector using the primer pair Fcab F1/ Fcab R1 (Table S3), digested with *Bsa*I and inserted it into the *Bsa*I cloning site of the magnICON®‐assembled vector pICHα31150 (Marillonnet *et al.*, 2005).

### Glyco‐modulating genes and other genes used in this investigation

Other binary vectors used in this investigation for the modulation of protein *N‐* and O‐glycosylation are described elsewhere: *Zea mays* core α1,3‐fucosyltransferases (α1,3‐FucT) and mouse core α1,6‐fucosyltransferases (α1,6‐FucT) (Castilho *et al.*, 2011a; Castilho *et al.*, 2015); human polypeptide *N‐*acetylgalactosaminyltransferase 2 (GalNAc‐T2) and *Drosophila melanogaster* core 1 β1,3‐galactosyltransferase (C1GALT1) (Castilho *et al.*, 2012); β1,4‐galactosyltransferase fused to the CTS region of the rat α2,6‐sialyltransferase under the Actin promoter (Act::^ST^GalT, Kallolimath *et al.*, 2018); P19 protein of *Tomato bushy stunt virus* (TBSV) (Garabagi *et al.*, 2012).

### NbBGAL1 cloning

A 160‐bp DNA fragment comprising the endogenous signal peptide of the *N. benthamiana* beta‐galactosidase 1 (*NbBGAL1*, NbS00024332g0007: 1‐72 nucleotides) flanked by M13 primer sequences, cohesive *Bsm*BI restriction sites and including *C*‐terminal *Bsa*I sites for further cloning was synthesized using GeneArt Strings DNA Fragments (https://www.thermofisher.com). The synthetic fragment was first amplified with M13 F/ M13 R primers (Table S3), digested with *Bsm*BI and cloned into *Bsa*I‐digested TMV‐based magnICON® vector (pICH26211; Marillonnet *et al.*, 2005). The resulting vector was named pICHβ26211.

The full length of *NbBGAL1* was commercially synthesized and previously cloned (Buscaill *et al.*, 2019). Here, the *NbBGAL1* cDNA (73‐2541 nucleotides) was amplified with flanking *Bsa*I restriction sites using the primer pairs BGAL F1/BGAL R1 (cDNA1) and BGAL1 F2/ BGAL1 R1 (cDNA2) that includes a *C*‐terminal sequence for a StrepII‐tag (WSHPQFEK) (Table S3). After *Bsa*I digestion, the cDNA1 was inserted into the *Bsa*I sites of pICHβ26211 and cDNA2 in the *Bsa*I cloning site of pICHα26211, containing the barley α‐amylase signal peptide (Schneider *et al.*, 2013). The resulting viral‐based vectors were termed SPβ‐BGAL1 and SPα‐BGAL1, respectively (Figure S1). To avoid virus competition and allow for co‐expression of *Nb*BGAL1 with reporter proteins already cloned in pICH26211α, we cloned *Nb*BGAL1 into pPT2 vector (Strasser *et al.*, 2007a). cDNA sequence from SPα‐BGAL1 was amplified out of the viral‐based vectors using the primer pair αSP F1/BGAL1 R3 (Table S3), digested with *Xba*I/*Bam*HI and ligated into pPT2 digested the same way (Figure S1). Additionally, the cDNA corresponding to the GH35 domain of the NbBGAL1 (73‐1080 nucleotides) was amplified with flanking *Bsa*I restriction sites using BGAL1 F2/ BGAL1 R2 primers (Table S3). After *Bsa*I digestion, the fragment was inserted into the *Bsa*I cloning site of pICHα26211 (SPα‐BGAL1‐GH35) (Figure S1).

To perform subcellular localization studies, *Nb*BGAL1 was *C*‐terminally tagged to the monomeric red fluorescent protein (mRFP) to generate SPβ‐BGAL1‐mRFP and SPα‐BGAL1‐mRFP fusions (Figure S1). Briefly, the cDNA sequences from SPβ‐BGAL1 and SPα‐BGAL1 were amplified out of the viral‐based vectors without the stop codon using the primer pairs βSP F1/BGAL1 R1 and αSP F1/BGAL1 R1, respectively (Table S3). PCR products were digested with *Xba*I/*Bam*HI and ligated into digested p31 (binary vector for expression of mRFP‐tagged proteins; Schoberer *et al.*, 2014).

### RNAiBGAL1 cloning

A RNA interference (RNAi) binary vector to knockdown the expression of *Nb*BGAL1 was generated in three cloning steps. First, the intron 2 of *A. thaliana* XYLT (Shin *et al.*, 2017) was amplified with primer pair intron F1/R1 introducing flanking *Xba*I‐*Xho*I and *Kpn*I‐*Bam*HI restriction sites. After *Xba*I/*Bam*HI digestion, the intron was ligated into pPT2 vector digested the same way. This resulted in a RNAi ‘preliminary’ binary vector (pRNAi). Next, a 290‐bp fragment corresponding to the coding sequence for amino acids 27‐111 of *Nb*BGAL1 was amplified using BGAL1 F3/ BGAL1 R4 primer pair creating *Xba*I‐*Bam*HI and *Xho*I‐*Kpn*I flanking restriction sites (Table S3). The PCR product digested *Bam*HI/*Kpn*I was cloned in *Kpn*I/*Bam*HI site of pRNAi generating an intron–antisense construct. Finally for the ‘sense’ fragment, PCR product was digested with *XbaI/XhoI* and inserted into *XbaI/XhoI* sites of the intron–antisense construct to generate a sense–intron–antisense hairpin vector (RNAiBGAL1; Figure S1).

### Transient expression, apoplastic fluid (AF) collection and protein purification

Recombinant proteins were transiently expressed in *N. benthamiana* leaves by agroinfiltration as described previously (Loos and Castilho, 2015). Agrobacteria containing magnICON® vectors were infiltrated at an optical density at 600 nm (OD_600_) of 0.1, while binary constructs were mixed at an OD_600_ of 0.05.

Isolation of secreted protein from the apoplastic fluid (AF) and total soluble protein (TSP) extraction were as before (Schneider *et al.*, 2015). Proteins were purified in small scale out of TSP by immunoaffinity with protein A as previously reported (Dicker *et al.*, 2015).

### SDS‐PAGE and immunoblotting

Proteins fractionated by 12% SDS‐PAGE under reducing conditions were either stained with Coomassie Brilliant Blue R‐250 or transferred to Hybond enhanced chemiluminescence nitrocellulose membranes (GE Healthcare) to be analysed by immunoblotting using specific antibodies/lectins.

‒ anti‐mRFP antibodies (6G6‐20 ChromoTek, 1:2000 in 5% milk/PBS‐Tween), detected using HRP‐conjugated anti‐mouse‐IgG (Promega, 1:10000 in 5% milk/PBS‐Tween).
‒ anti‐Lewis a antibodies (1:400 JIM84 in 3% milk/PBS‐Tween, provided by Richard Strasser, BOKU, Vienna), detected using HRP‐conjugated anti‐rat IgM (Jackson, 1:10000 in PBS‐Tween).
‒ lectin blotting using Biotinylated *Ricinus Communis Agglutinin* I (RCA, Vector, 5‐10 µg/mL in 3% BSA, biotin‐free/PBS‐Tween), detected using HRP‐conjugated Streptavidin (1mg/mL, Vector, 1:8000 in PBS‐Tween).

Clarity™ Western enhanced chemiluminescence reagents (BIO‐RAD Laboratories, Inc., Hercules, CA, USA) were used as substrates. Finally, membranes blots were stained with Ponceau S (Sigma Aldrich, St. Louis, MO, USA) to visualize transferred proteins.

### Confocal imaging of fluorescent protein fusions

Leaves of 4‐ to 5‐week‐old *N. benthamiana* plants were infiltrated with agrobacterium suspensions carrying binary plant expression vectors for mRFP‐tagged proteins together with a plasma membrane marker EGFP‐LTI6b (PM‐GFP; Strasser *et al.*, 2007b).

High‐resolution images were acquired 2 days post‐infiltration (dpi) on an upright Leica SP5 II confocal microscope using the Leica LAS AF software system. mRFP was excited with 561‐nm laser and detected at 600–630 nm. Post‐acquisition image processing was performed in Adobe Photoshop cs5.

### *In vitro* β‐galactosidase activity assay and NbBGAL1 kinetics

Fluorescein di‐β‐D‐galactopyranoside (FDG) was first dissolved in ethanol + DMSO (1:1) and stored as 2 mm stock solution in DMSO + ethanol+water (1:1:8). Eighty microlitres of AF isolated from *N. benthamiana* leaves was incubated for one hour at 25°C with 0.2 µm FDG and 50 mm MES buffer with pH 5 in the total reaction volume of 100 µL. The fluorescence of fluorescein, a product of FDG hydrolysis by β‐galactosidase, was measured after one hour using a 96‐well plate reader (1420 multilabel counter Victor^2^_TM_ Wallac Oy) with an excitation wavelength of 485 nm and emission wavelength of 535 nm. A β‐galactosidase purified from *Aspergillus oryzae* with a known concentration (276 U/mL) was used as a reference to quantify β‐galactosidase activity in 1 mL of AF.

The effect of pH on the activity of *Nb*BGAL1 was determined by FDG assay, where AF‐derived SPα‐BGAL1 was incubated with 0.2 µm FDG at 25°C for one hour in 50 mm MES buffer with pH ranging from 2.0 to 9.0. Similarly, the effect of metal ions on the activity of *Nb*BGAL1 was examined by FDG assay by incubating the AF‐derived SPα‐BGAL1 in 50 mm MES buffer supplemented with 5 mm of FeCl_2_, CaCl_2_, CuSO_4_ and EDTA.

*Nb*BGAL1 *in vitro* activity was also assayed by incubating reporter proteins carrying terminal galactosylated glycans (IgG, A1AT, TF and Fcab) overnight at 37°C with AF from *N. benthamiana* and with AF from plants overexpressing SPα‐BGAL1.

The effect of temperature on the activity of *Nb*BGAL1 was examined by incubating galactosylated A1AT with AF‐derived SPα‐BGAL1 at different temperatures (25, 37, 50, 60 and 95 °C). Fully galactosylated reporter proteins were generated by digestion of sialic acid residues with neuraminidase (New England Biolabs) according to the manufacturer’s instructions. Commercially available human TF and A1AT are natural sialylated, and sialylated Fcab‐Her2 was produced by co‐expressing the reporter protein in ΔXTFT^SIA^ plants (Kallolimath *et al.*, 2016) with α1,3‐FucT, which is used to enhance sialylation of plant‐derived Fc (Castilho *et al.*, 2015).

Kinetic parameters of AF‐derived SPα‐BGAL1 were estimated by incubation AF‐derived SPα‐BGAL1 with various concentrations of FDG in 50 mM MES (pH 5.0) at 25°C for one hour. Km and Vmax were calculated from the Hanes–Woolf and Lineweaver–Burk plots of the data at 8 different concentrations of FDG ranging from 0 to 250 µM. The values represent the means of three independent experiments.

### Glycosidase activity profiling

The activity of glycosidases on the secretome of *N. benthamiana* was evaluated by activity‐based protein profiling (ABPP) as described previously (Buscaill *et al.*, 2019).

### Glycan analysis

*N‐*glycosylation profile of TSP extracts from *N. benthamiana* leaves was determined by matrix‐assisted laser desorption/ionization time‐of‐flight mass spectrometry (MALDI‐TOF) of *N‐*glycans released from glycopeptides by peptide *N‐*glycosidase A as described previously (Kolarich *et al.*, 2015). Site‐specific *N‐*glycosylation profiling of recombinant proteins was determined using reversed‐phase liquid chromatography–electrospray ionization mass spectrometry (LC‐ESI‐MS) of tryptic glycopeptides as described previously (Gruber and Altmann, 2015). For EpoFc, a double digestion with trypsin and endoproteinase Glu‐C allows site‐specific analysis of all three Epo *N‐*glycosylation sites.

## Conflict of interest

The authors declared that they have no conflict of interests.

## Author contributions

RK and EZ performed experiences. CGG performed glycan analysis. PB and RALH wrote manuscript and provided scientific support and BGAL1 cDNA sequence. AC designed experiments and wrote the manuscript.

## Supporting information


**Figure S1** Binary vectors for modulation of *Nb*BGAL1 expression in* Nicotiana benthamiana.*
**Figure S2** Coomassie staining of secreted proteins (AF) in *N. benthamiana *plants expressing *Nb*BGAL1.
**Figure S3** Site‐specific *N*‐glycosylation profile of *Nb*BGAL1 expressed in ΔXTFT^GAL^.
**Figure S4** *N*‐glycosylation profiles of different reporter glycoproteins expressed ΔXTFT^GAL^.
**Figure S5** *N*‐glycosylation profile of an IgG1‐Fc expressed ΔXTFT^GAL^ in the presence or absence of core‐fucose.
**Figure S6** *N*‐glycosylation profiles of human A1AT (hA1AT) and plant‐derived Fcab‐Her2 (Fcab).
**Figure S7** *N*‐glycosylation profiles of Fcab‐Her2 (Fcab) and EpoFc co‐expressed in ΔXTFT^GAL^ without (‐) or with (+) SPα‐BGAL1.
**Figure S8** *Nb*BGAL1 removes terminal galactose residues from endogenous glycoproteins.
**Figure S9** *N*‐glycosylation profile of EpoFc co‐expressed in *N. benthamiana *WT plants without (‐) or with (+) SPα‐BGAL1.
**Figure S10** Generation of the T‐antigen (Galβ1‐3GalNAc‐) on recombinant plant‐produced EpoFc.
**Figure S11** *N*‐glycosylation profiles of Cx‐IgG‐Fab, Fcab‐Her2 (Fcab) and EpoFc co‐expressed in ΔXTFT^GAL^ without (‐) or with (+) RNAiBGAL1.
**Figure S12** *N*‐glycosylation profiles of Cx‐IgG‐Fab, Fcab‐Her2 (Fcab), TF and EpoFc co‐expressed with ^ST^GalT in the *N. benthamiana *wild type (WT) or in mutant plants (*bgal1‐1*).
**Figure S13** Schematic representation of the *N‐* and *O*‐glycan structures identify by LC‐ESI‐MS on recombinant glycoproteins expressed during this investigation.
**Table S1** Peptide sequence and mass of glycosylation sites (GP) present in different reporter glycoproteins used in this investigation.
**Table S2** Relative abundance (%) of β1,4‐galactosylated *N‐*glycans on selected reporter proteins.
**Table S3** Sequence of primers used in this study.
