# Supplementary material for: BGAL1 depletion boosts the level of β‐galactosylation of N‐ and O‐glycans in N. benthamiana
Source: Plant Biotechnol J. 2020 Jan 11;18(7):1537–49. doi: 10.1111/pbi.13316 (PMC7292537; doi:10.1111/pbi.13316)
Supplement: Supplementary file 1 — Figure S1 Binary vectors for modulation of NbBGAL1 expression in Nicotiana benthamiana. Figure S2 Coomassie staining of secreted proteins (AF) in N. benthamiana plants expressing NbBGAL1. Figure S3 Site‐specific N‐glycosylation profile of NbBGAL1 expressed in ΔXTFTGAL. Figure S4 N‐glycosylation profiles of different reporter glycoproteins expressed ΔXTFTGAL. Figure S5 N‐glycosylation profile of an IgG1‐Fc expressed ΔXTFTGAL in the presence or absence of core‐fucose. Figure S6 N‐glycosylation profiles of human A1AT (hA1AT) and plant‐derived Fcab‐Her2 (Fcab). Figure S7 N‐glycosylation profiles of Fcab‐Her2 (Fcab) and EpoFc co‐expressed in ΔXTFTGAL without (‐) or with (+) SPα‐BGAL1. Figure S8 NbBGAL1 removes terminal galactose residues from endogenous glycoproteins. Figure S9 N‐glycosylation profile of EpoFc co‐expressed in N. benthamiana WT plants without (‐) or with (+) SPα‐BGAL1. Figure S10 Generation of the T‐antigen (Galβ1‐3GalNAc‐) on recombinant plant‐produced EpoFc. Figure S11 N‐glycosylation profiles of Cx‐IgG‐Fab, Fcab‐Her2 (Fcab) and EpoFc co‐expressed in ΔXTFTGAL without (‐) or with (+) RNAiBGAL1. Figure S12 N‐glycosylation profiles of Cx‐IgG‐Fab, Fcab‐Her2 (Fcab), TF and EpoFc co‐expressed with STGalT in the N. benthamiana wild type (WT) or in mutant plants (bgal1‐1). Figure S13 Schematic representation of the N‐ and O‐glycan structures identify by LC‐ESI‐MS on recombinant glycoproteins expressed during this investigation. Table S1 Peptide sequence and mass of glycosylation sites (GP) present in different reporter glycoproteins used in this investigation. Table S2 Relative abundance (%) of β1,4‐galactosylated N‐glycans on selected reporter proteins. Table S3 Sequence of primers used in this study. [file PBI-18-1537-s001.docx]

**Supplementary data**

**Table S1:** Peptide sequence and mass of glycosylation sites (GP) present in different reporter glycoproteins used in this investigation. Fcab-Her2: Fc fragment with engineered HER2/neu-binding sites; Cx-IgG: monoclonal antibody Cetuximab; EpoFc: erythropoietin fused to an Fc fragment TF: human transferrin; A1AT: human alpha-1 anti-trypsin and *Nb*BGAL1: *Nicotiana benthamiana* β-galactosidase 1. Da, Dalton

| **Protein** | **Glycopeptide (GP)** | **Mass (Da)** |
| --- | --- | --- |
| Fcab-HER2 | GP1 (Fc): EEQYNSTYR | 1189.51 |
| Cx-IgG | GP1 (Fab): MNSLQSNDTAIYYCAR | 1907.80 |
|  | GP2 (Fc): EEQYNSTYR | 1189.51 |
| EpoFc | GP1: EAENITTGCAE | 1194.49 |
|  | GP2: HCSLNENITVPDTK | 1627.77 |
|  | GP3: GQALLVNSSQPWEPLQLHVDK | 2359.24 |
| TF | GP1: GLVPVLAENYNKSDNCEDTPEAGYFAVAVVKK | 1476.75 |
|  | GP2: QQHLFGSNVTDCSGNFCLFR | 2515.10 |
| A1AT | GP1: QLAHQSNSTNIFFSPVSIATAFAMLSLGTK | 3181.00 |
|  | GP2: ADTHDEILEGLNFNLTEIPEAQIHEGFQELLR | 3691.82 |
|  | GP3: YLGNATAIFFLPDEGK | 1755.89 |
| *Nb*BGAL1 | GP1:ANVTYDHR | 975.46 |
|  | GP3:TGLMEQINTTADK | 1421.69 |
|  | GP4: NGSTIDLSSK | 1021.52 |

**Table S2**: Relative abundance (%) of β1,4-galactosylated *N-*glycans on selected reporter proteins. *In vitr*o activity of endogenous and recombinant *Nb*BGAL1 was assessed by determining the levels of di-galactosylated *N-*glycans (AA) on reporter proteins before and after *in vitro* incubations with *Nb*BGAL1 naturally present in plant apoplast (AF) or over-expressed in *N. benthamiana* plants (AF+SPα-BGAL1). For detailed glycoprofiles see Figure S6.

| ***In vitro* activity** | | |
| --- | --- | --- |
| **IgG** | **Incubation** | **AA (%)** |
| Fc | - | 48 |
|  | AF | 34 |
|  | AF+SPα-BGAL1 | 13 |
| **A1AT** | **Incubation** | **AA (%)** |
| GP1 | - | 100* |
|  | AF | 31.5 |
|  | AF+SPα-BGAL1 | 13.6 |
| GP2 | - | 100* |
|  | AF | 55.8 |
|  | AF+SPα-BGAL1 | 15.2 |
| GP3 | - | 100* |
|  | AF | 80.5 |
|  | AF+SPα-BGAL1 | 12.9 |
| **Fcab+α1,3-FucT** | **Incubation** | **AAF^3^ (%)** |
| Fc | - | 82* |
|  | AF | 51 |
|  | AF+SPα-BGAL1 | 17 |

*Di-galactosylated glycans (AA) were obtained by digestion of terminal sialic acids residues

**Table S3**: Sequence of primers used in this study

| **Primer** | **Restriction** | **Sequence (5´-3`)** |
| --- | --- | --- |
| GalT1 F1 | *Xba*I | TATA*TCTAGA*ATGAAGAGATTTTATGGAGG |
| GalT1 R1 | *BamHI* | TATA*GGTACC*AAAGTCAGGGAGAGTAATCCG |
| Fcab F1 | *BsaI* | GCA*GGTCTC*AAGGTatggAGCTGGGCCTGAGCTGG |
| Fcab R1 | *BsaI* | GCA*GGTCTC*AAAGCTCATTTACCCGGAGACAGGG |
| M13F | - | GTAAAACGACGGCCAG |
| M13R | - | CAGGAAACAGCTATGAC |
| BGAL1 R1 | *Bsa*I-Strep-*Bam*HI | TATA*GGTCTC*AAAGCTTA**cttttcgaactgcggatggctcca***ggatcc*CGAACAGGAAGCTTCTACGGC |
| BGAL1 R2 | *Bsa*I-Strep-*Bam*HI | TATA*GGTCTC*AAAGCTTA**cttttcgaactgcggatggctcca***ggatcc*AGATCCTGGAGAAGTGATAG |
| BGAL1 R3 | *BamHI* | TATA*ggatcc*TTACGAACAGGAAGCTTCTACGGC |
| BGAL F1 | *BsaI* | GCA*GGTCTC*AAGGTCCGCCAATGTGACGTATGATCACC |
| BGAL F2 | *BsaI* | GCA*GGTCTC*AAGGTGCCAATGTGACGTATGATCACC |
| βSP F1 | XbaI | TATA*TCTAGA*ATGATGGGTTTATCCGTAATGC |
| αSP F1 | *XbaI* | TATA*TCTAGA*ATGGCGAACAAACACTTGTCCCTCTCCCTCTT |
| Intron F1 | *XbaI-XhoI* | TATA*TCTAGACTCGAG*acactgcacggtatgctcc |
| Intron R1 | *BamHI-KpnI* | TATA*GGATCCGGTACC*ctagctgcgtctgcaaaaagattc |
| BGAL1 F3 | *XbaI-BamHI* | TATA*TCTAGAggatcc*GCCAATGTGACGTATGATCACCG |
| BGAL1 R4 | *Xho*I-*Kpn*I | TATA*CTCGAGGGTACC*GCACAAACATAAGGCCCGATC |

Restriction sites are in italic and underlined. Strep-tag sequence is in bold.

**
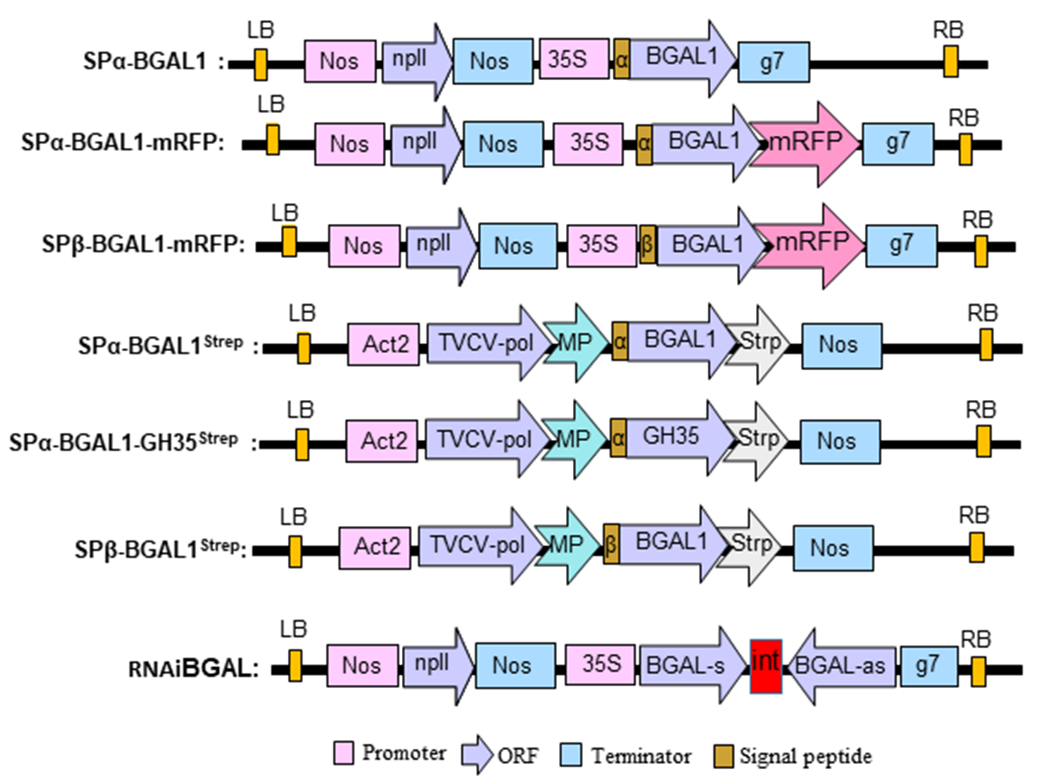
**

**Figure S1: Binary vectors for modulation of *Nb*BGAL1 expression in *Nicotiana benthamiana.*** Schematic representation of the binary vectors used to express the galactosidase gene (*Nb*BGAL1, NbS00024332g0007.1). *Nb*BGAL1 was targeted to the secretory pathway using either the barley α-amylase signal peptide (α) or the *Nb*BGAL1 endogenous signal peptide (β). The cDNA was cloned in common binary vectors or in Tobacco Mosaic Virus (TMV)-based vectors (pICH26211) carrying either the β or the α signal peptide. *Nb*BGAL1 was *C*-terminally tagged with StrepII-tag to assess protein expression and with mRFP for subcellular localisation by confocal laser scanning microscopy. To down regulate the expression of *Nb*BGAL1 a 290 bp fragment corresponding to the coding sequence for amino acids 27-111 was cloned in sense and antisense orientation spaced by an intron to generate a RNA interference construct (RNAiBGAL1).

35S: cauliflower mosaic virus (CaMV) 35S promoter; Act2: Arabidopsis actin 2 promoter; *Nb*BGAL1: cDNA from the *N. benthamiana* beta-galactosidase (25-846 aa); g7: *Agrobacterium* gene 7 terminator; GH35: catalytic domain of the *Nb*BGAL1 from the Glycosyl Hydrolase 35 family (25-360 aa); int: intron derived from *A. thaliana* β1,2-xylosyltransferase; LB: left border; MP: movement protein from TMV; mRFP: monomeric red fluorescent protein; Nos: nopaline synthase promoter or terminator; npII: neomycin phosphotransferase II gene conferring resistance to kanamycin; RB: right border; Strp: StrepII-tag (WSHPQFEK peptide); TVCV-Pol: RNA-dependent RNA polymerase from turnip vein clearing virus; α: signal peptide from barley alpha amylase (1-24 aa); β: signal peptide from the *N. benthamiana* BGAL1 (1-24 aa).

**
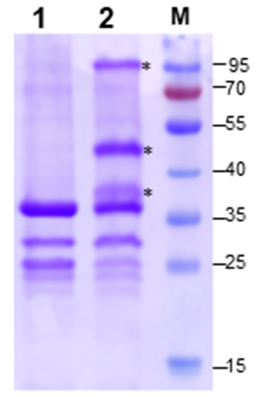
**

**Figure S2: Coomassie staining of secreted proteins (AF) in *N. benthamina* plants expressing *Nb*BGAL1.** (1) SPα-BGAL1-GH35 (2) SPα-BGAL1. Protein bands identified as *Nb*BGAL1 by peptide mapping are marked (*). Protein size marker (M) is shown in kilo Dalton (kDa).

**
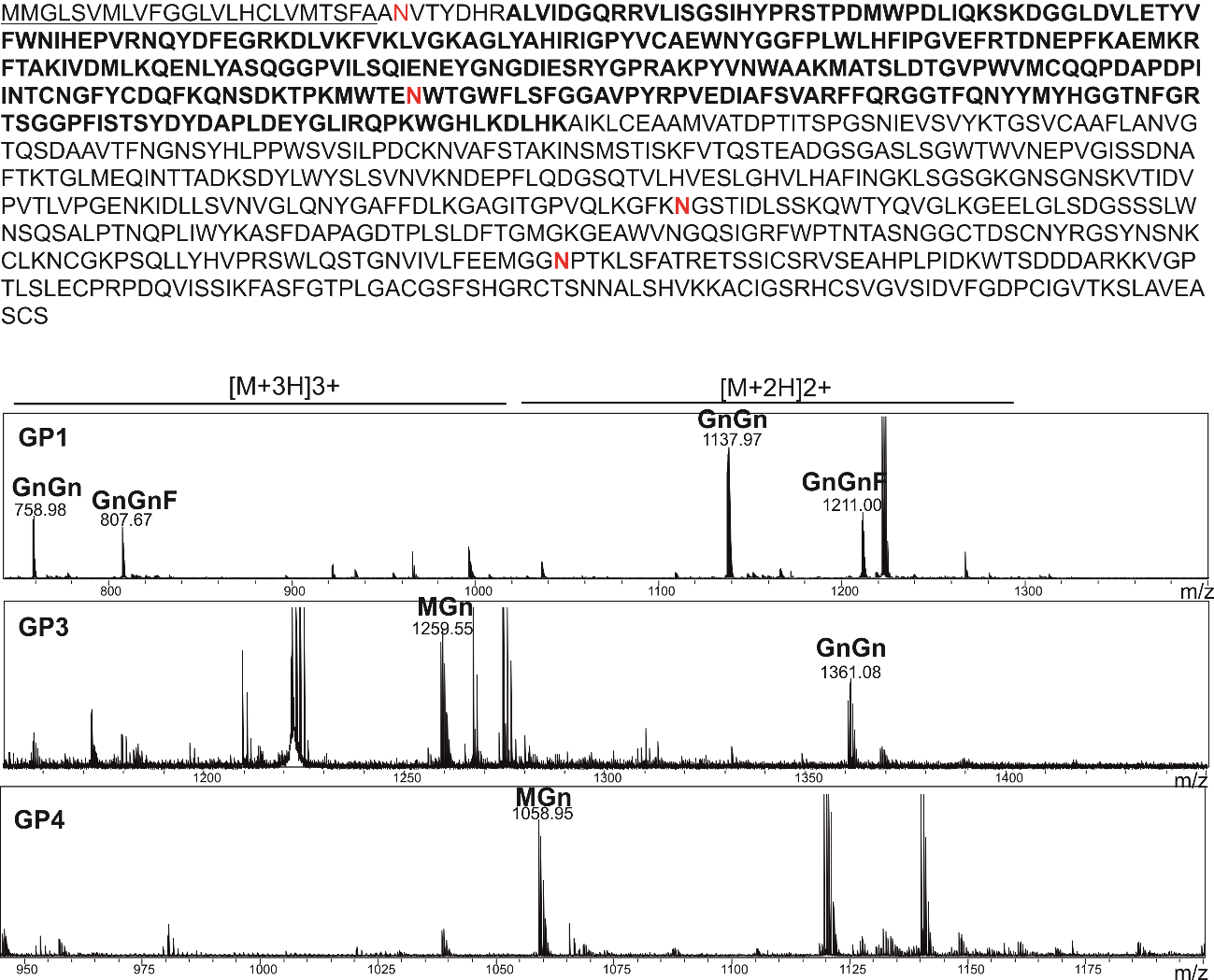
**

**Figure S3: Site-specific *N*-glycosylation profile of *Nb*BGAL1 expressed in ΔXTFT^GAL^.** (Top) *Nb*BGAL1 peptide sequence shows endogenous (β) signal peptide (underlined), GH35 domain (bold) and potential glycosylation sites (red). (Bottom) *N-*glycosylation profiles of tryptic digested glycopeptides GP1, 3 and 4 (Table S1) determined by LC-ESI-MS. The major glycosylated peaks [M+2H]^2+^ and [M+3H]^3+^ are depicted. Symbol nomenclature in accordance with the Consortium of Functional Glycomics (http://glycomics.scripps.edu/CFGnomenclature.pdf). For interpretation of glycoforms present in assigned peaks see Figure S13.

**
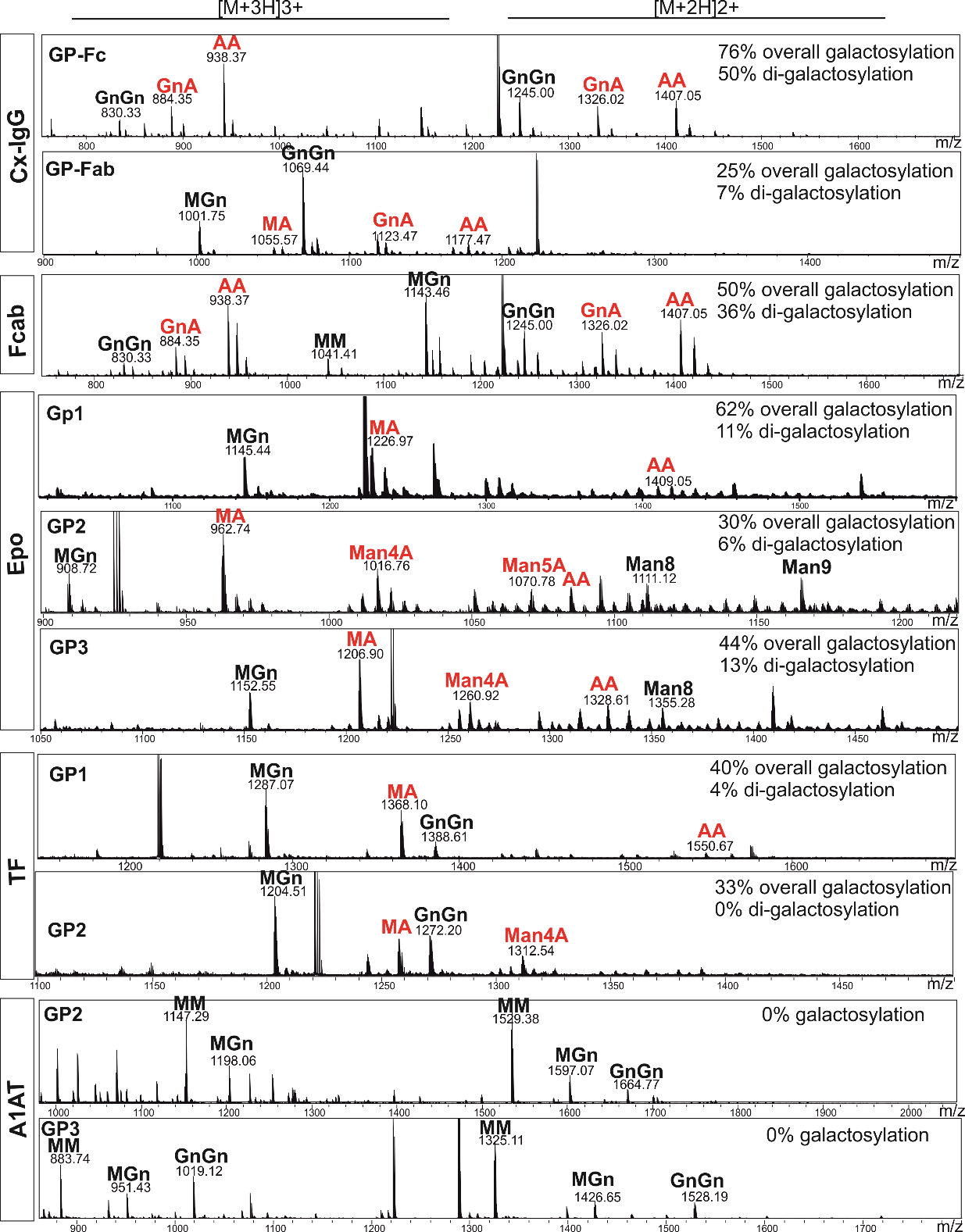
**

**Figure S4: *N*-glycosylation profiles of different reporter glycoproteins expressed ΔXTFT^GAL^.** Glycan profiles of tryptic (or trypsin+GluC) digested glycopeptides (Table S1) were determined by LC-ESI-MS. The major glycosylated peaks [M+2H]^2+^ and [M+3H]^3+^ are depicted. Symbol nomenclature in accordance with the Consortium of Functional Glycomics (http://glycomics.scripps.edu/CFGnomenclature.pdf). For interpretation of glycoforms present in assigned peaks see Figure S13.


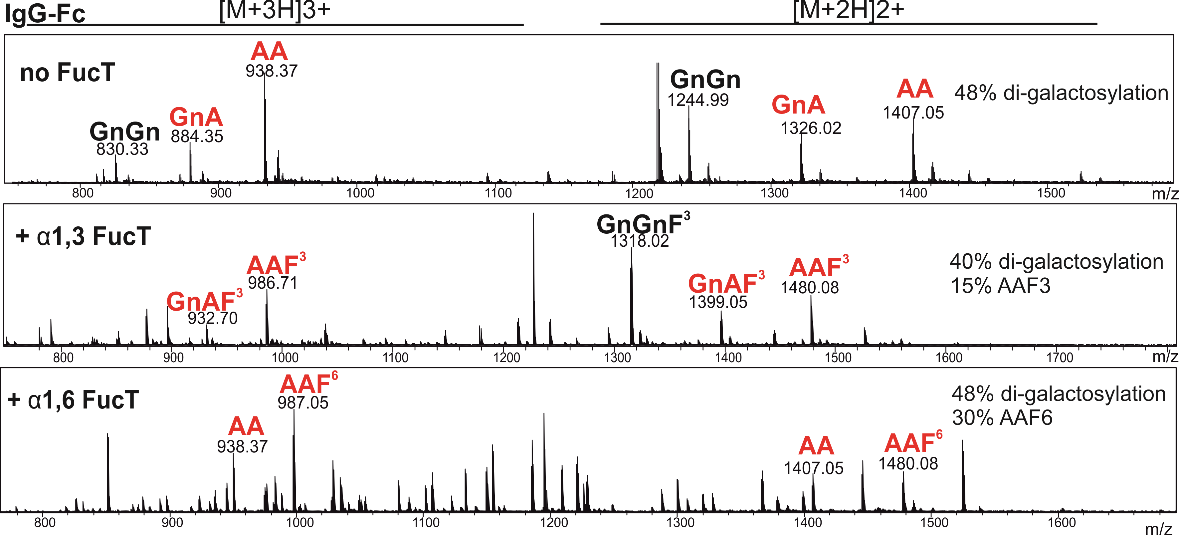


**Figure S5: *N*-glycosylation profile of an IgG1-Fc expressed ΔXTFT^GAL^** **in the presence or absence of core-fucose.** Cx-IgG was transiently co-expressed without or with plant-(α1,3-FucT) and mammalian-(α1,6-FucT) core fucosyltransferases. Glycan profiles of tryptic digested Fc-glycopeptide (Table S1) were determined by LC-ESI-MS. The major glycosylated peaks [M+2H]^2+^ and [M+3H]^3+^ are depicted. Symbol nomenclature in accordance with the Consortium of Functional Glycomics (http://glycomics.scripps.edu/CFGnomenclature.pdf). For interpretation of glycoforms present in assigned peaks see Figure S13.


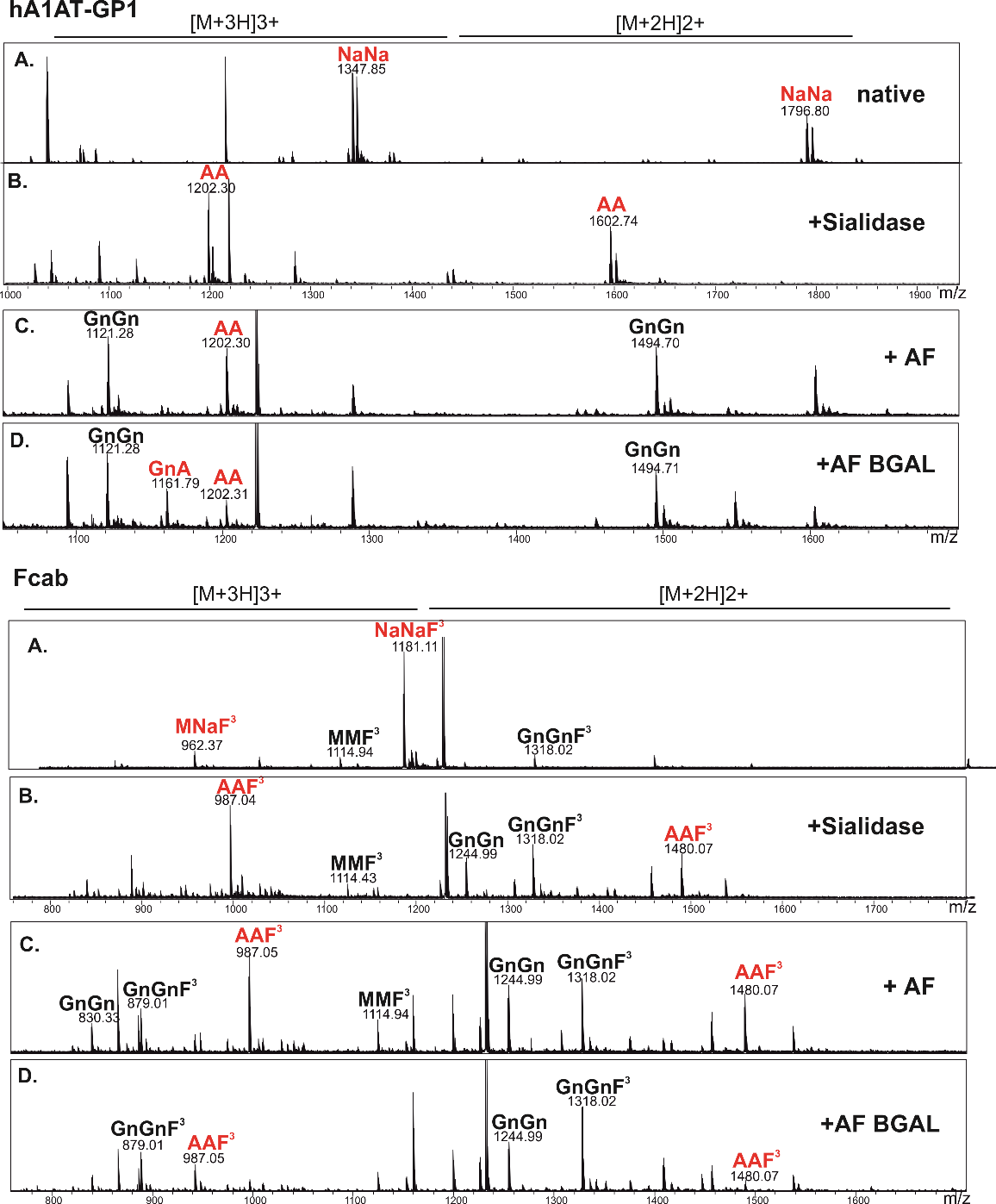


**Figure S6: *N*-glycosylation profiles of human A1AT (hA1AT) and plant-derived Fcab-Her2 (Fcab). A.** Plasma-A1AT (top) and recombinant Fcab produced in ΔXTFT^SIA^ plants (bottom) show high levels of sialylated *N-*glycans. **B.** Sialidase treatment of hA1AT (Top) and Fcab (Bottom) removes terminal sialic acids and exposes β1,4-galactose residues. **C**. and **D.** *In vitro* incubation of hA1AT (Top) and Fcab (Bottom) with AF collected from *N. benthamiana* WT (+AF) or with AF collected from WT *N. benthamiana* expressing SPα-BGAL1 (+AF BGAL) drastically reduces the levels of di-galactosylation *N-*glycans. Glycan profiles of tryptic digested glycopeptides (Table S1) were determined by LC-ESI-MS. The major glycosylated peaks [M+2H]^2+^ and [M+3H]^3+^ are depicted. Symbol nomenclature in accordance with the Consortium of Functional Glycomics (http://glycomics.scripps.edu/CFGnomenclature.pdf). For interpretation of glycoforms present in assigned peaks see Figure S13.

**
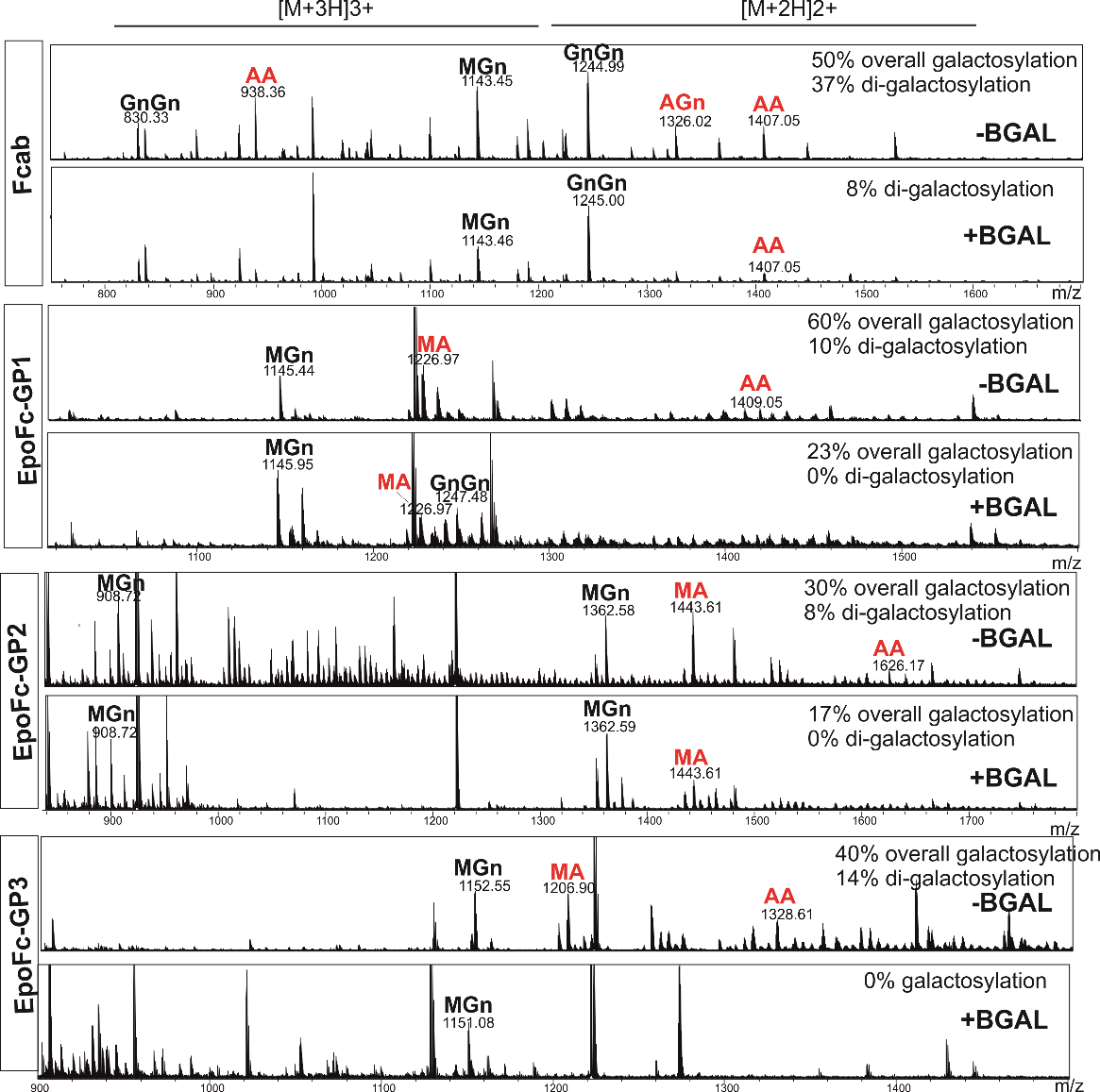
**

**Figure S7: *N*-glycosylation profiles of Fcab-Her2 (Fcab) and EpoFc co-expressed in ΔXTFT^GAL^ without (-) or with (+) SPα-BGAL1.** Glycan profiles of tryptic (or trypsin +GluC) digested glycopeptides (Table S1) were determined by LC-ESI-MS. Relative abundance of di-galactosylated (AA) glycans is shown. The major glycosylated peaks [M+2H]^2+^ and [M+3H]^3+^ are depicted. Symbol nomenclature in accordance with the Consortium of Functional Glycomics (http://glycomics.scripps.edu/CFGnomenclature.pdf). For interpretation of glycoforms present in assigned peaks see Figure S13.

**
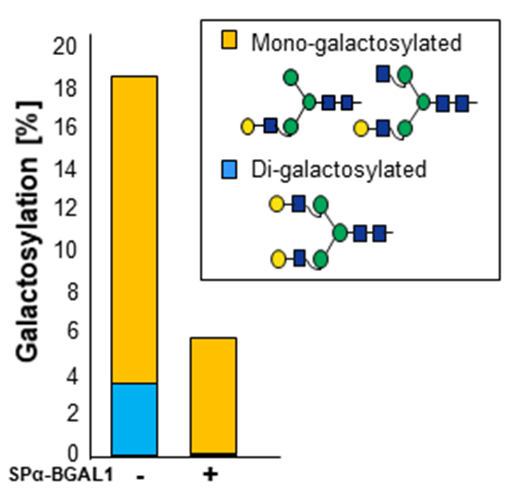
**

**Figure S8: *Nb*BGAL1 removes terminal galactose residues from endogenous glycoproteins.** Relative abundance (%) of β1,4-galactosylated (mono- and di-antennary) *N*-glycans on secreted endogenous proteins present in AF of ∆XTFT^GAL^ plants without (-) or with (+) overexpression of SPα-BGAL1.

**
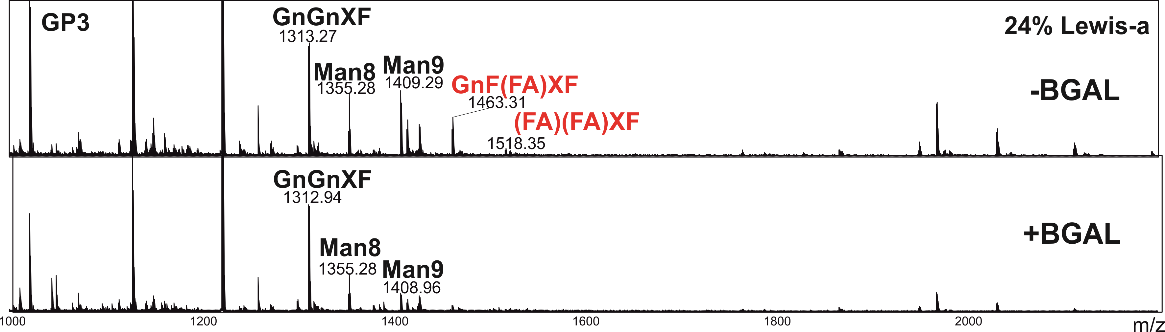
**

**Figure S9: *N*-glycosylation profile of EpoFc co-expressed in *N. benthamiana* WT plants without (-) or with (+) SPα-BGAL1.** The synthesis *N-*glycans carrying mono- or di-antennary Lewis-A structures (Top) is completely inhibited by the co-expression of SPα-BGAL1 (bottom). Epo GP3 (Table S1) is shown as a representative. Relative abundance of *N-*glycans with Lewis-A epitopes is shown. The major glycosylated peaks [M+2H]^2+^ are depicted. Symbol nomenclature in accordance with the Consortium of Functional Glycomics (http://glycomics.scripps.edu/CFGnomenclature.pdf). For interpretation of glycoforms present in assigned peaks see Figure S13.


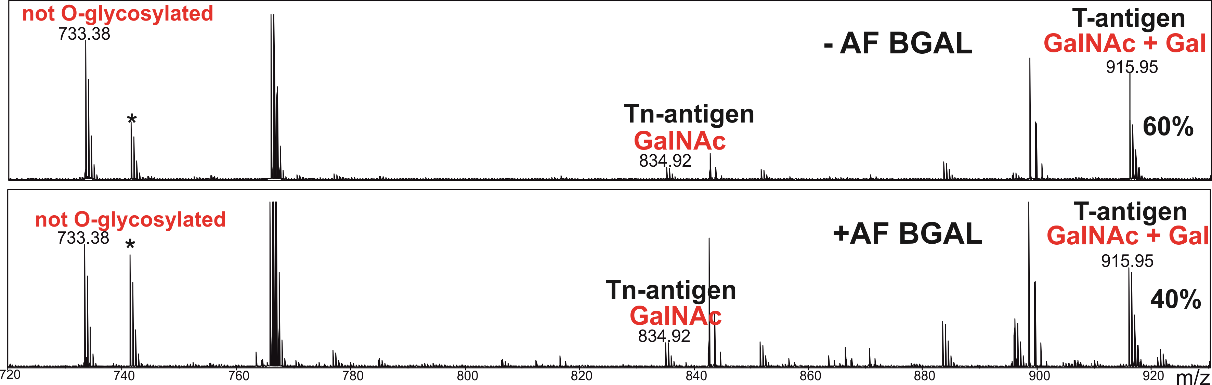


**Figure S10: Generation of the T-antigen (Galβ1-3GalNAc-) on recombinant plant-produced EpoFc.** EpoFc was co-expressed in *N. benthamiana* with the machinery for the synthesis of mucin-type *O-*glycans. The generation of T-antigen (GalNAc+Gal) on Ser-126 containing peptide (^117^EAISPPDAASAAPLR^131^) was monitored by LC-ESI-MS. *O*-glycosylated EpoFc was purified and incubated with either PBS or with AF isolated from *N. benthamiana* expressing SPα-BGAL1 (+AF BGAL) for 1 and 4 hours. The levels of T-antigen were compared to non-*O*-glycosylated EpoFc. Nomenclature in accordance with the Consortium of Functional Glycomics (http://glycomics.scripps.edu/CFGnomenclature.pdf). For interpretation of glycoforms present in assigned peaks see Figure S13.

**
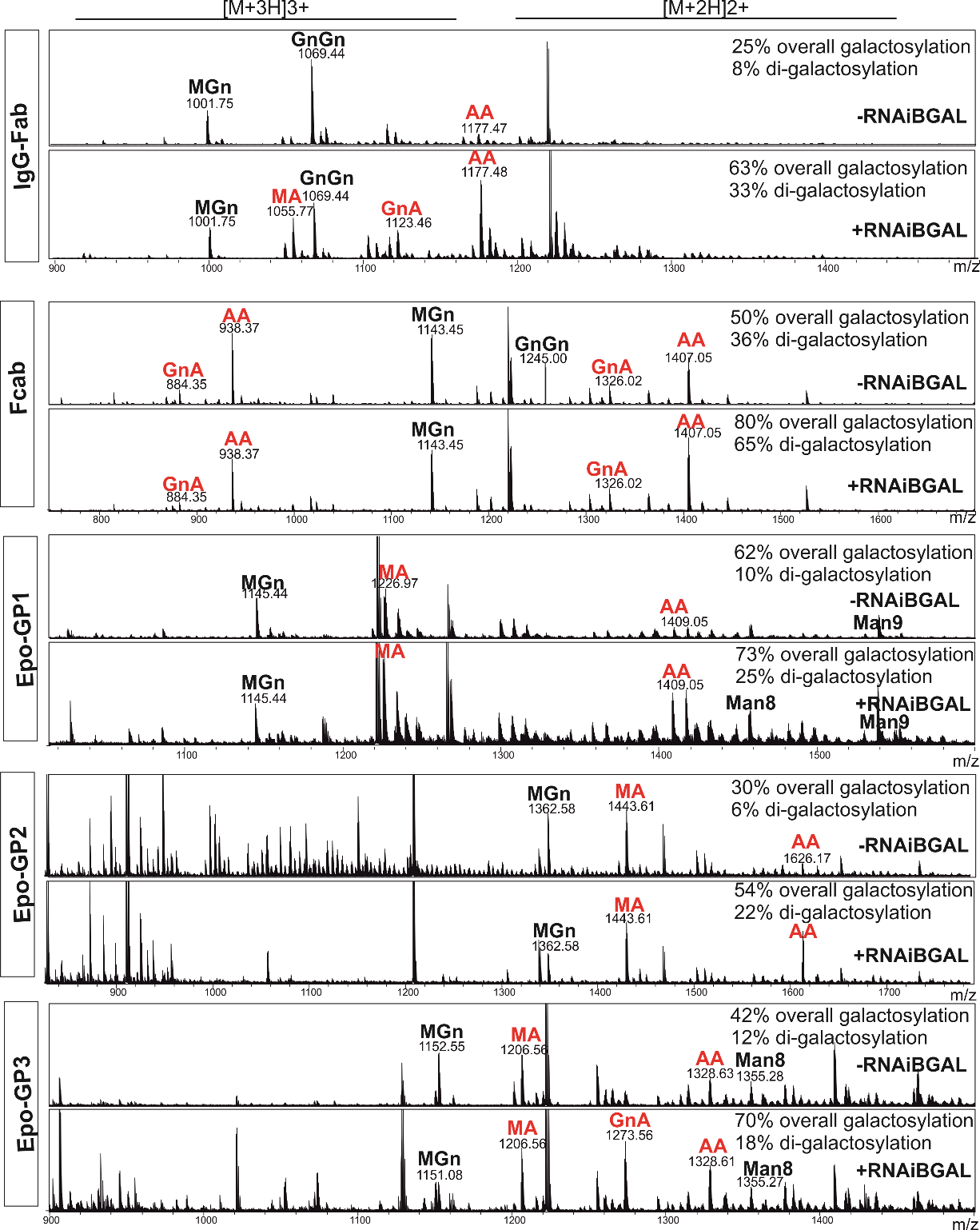
**

**Figure S11: *N*-glycosylation profiles of Cx-IgG-Fab, Fcab-Her2 (Fcab) and EpoFc co-expressed in ΔXTFT^GAL^ without (-) or with (+) RNAiBGAL1.** Glycan profiles of tryptic (or trypsin +GluC) digested glycopeptides (Table S1) were determined by LC-ESI-MS. Relative abundance of di-galactosylated (AA) glycans is shown. The major glycosylated peaks [M+2H]^2+^ and [M+3H]^3+^ are depicted. Symbol nomenclature in accordance with the Consortium of Functional Glycomics (http://glycomics.scripps.edu/CFGnomenclature.pdf). For interpretation of glycoforms present in assigned peaks see Figure S13.

**
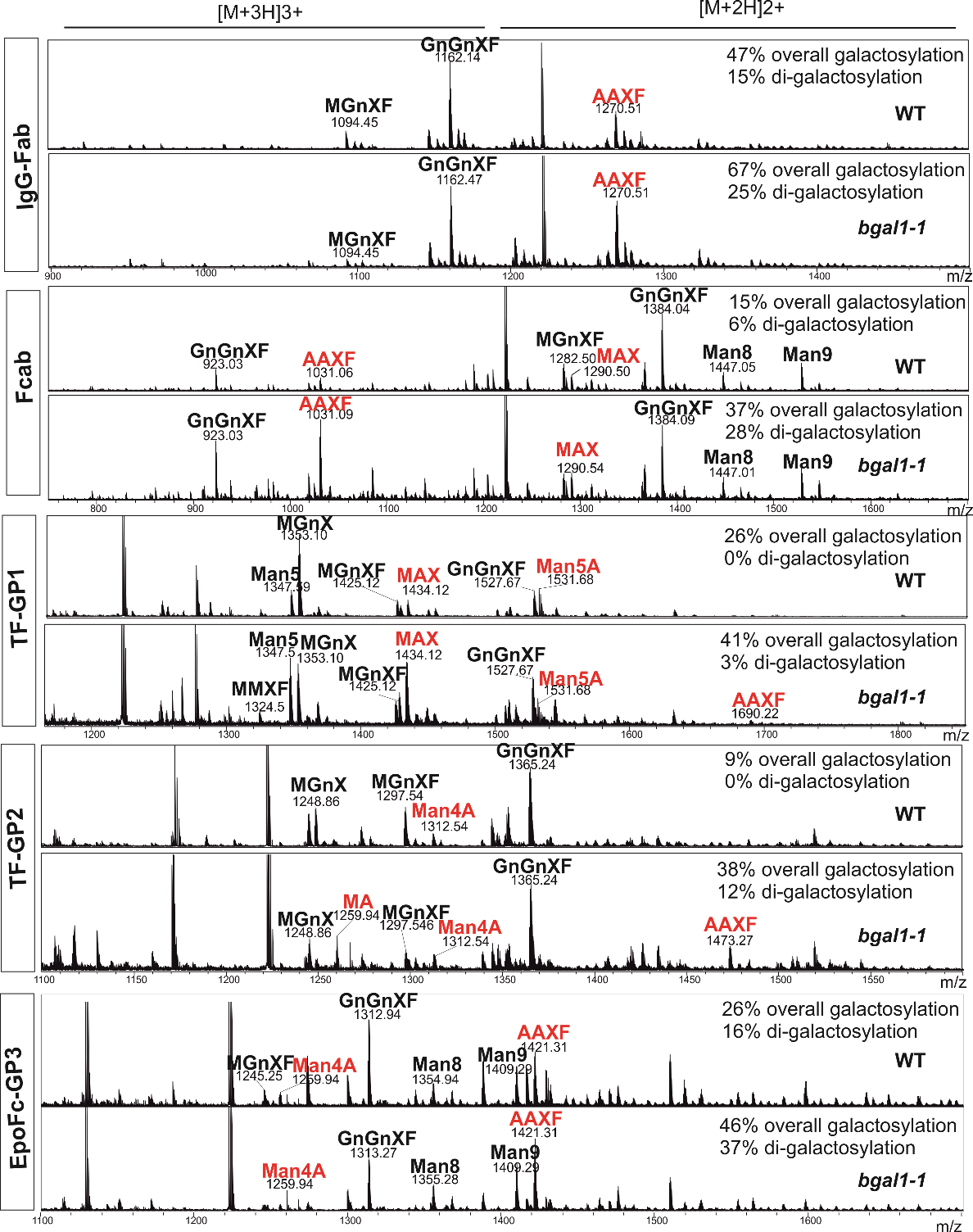
**

**Figure S12: *N*-glycosylation profiles of Cx-IgG-Fab, Fcab-Her2 (Fcab), TF and EpoFc co-expressed with ^ST^GalT in the *N. benthamiana* wild type (WT) or in mutant plants (*bgal1-1*).** Glycan profiles of representative tryptic (or trypsin +GluC) digested glycopeptides (Table S1) were determined by LC-ESI-MS. Relative abundance (%) of di-galactosylated (AAXF) glycans is shown. The major glycosylated peaks [M+2H]^2+^ and [M+3H]^3+^ are depicted. Symbol nomenclature in accordance with the Consortium of Functional Glycomics (http://glycomics.scripps.edu/CFGnomenclature.pdf). For interpretation of glycoforms present in assigned peaks see Figure S13.

**
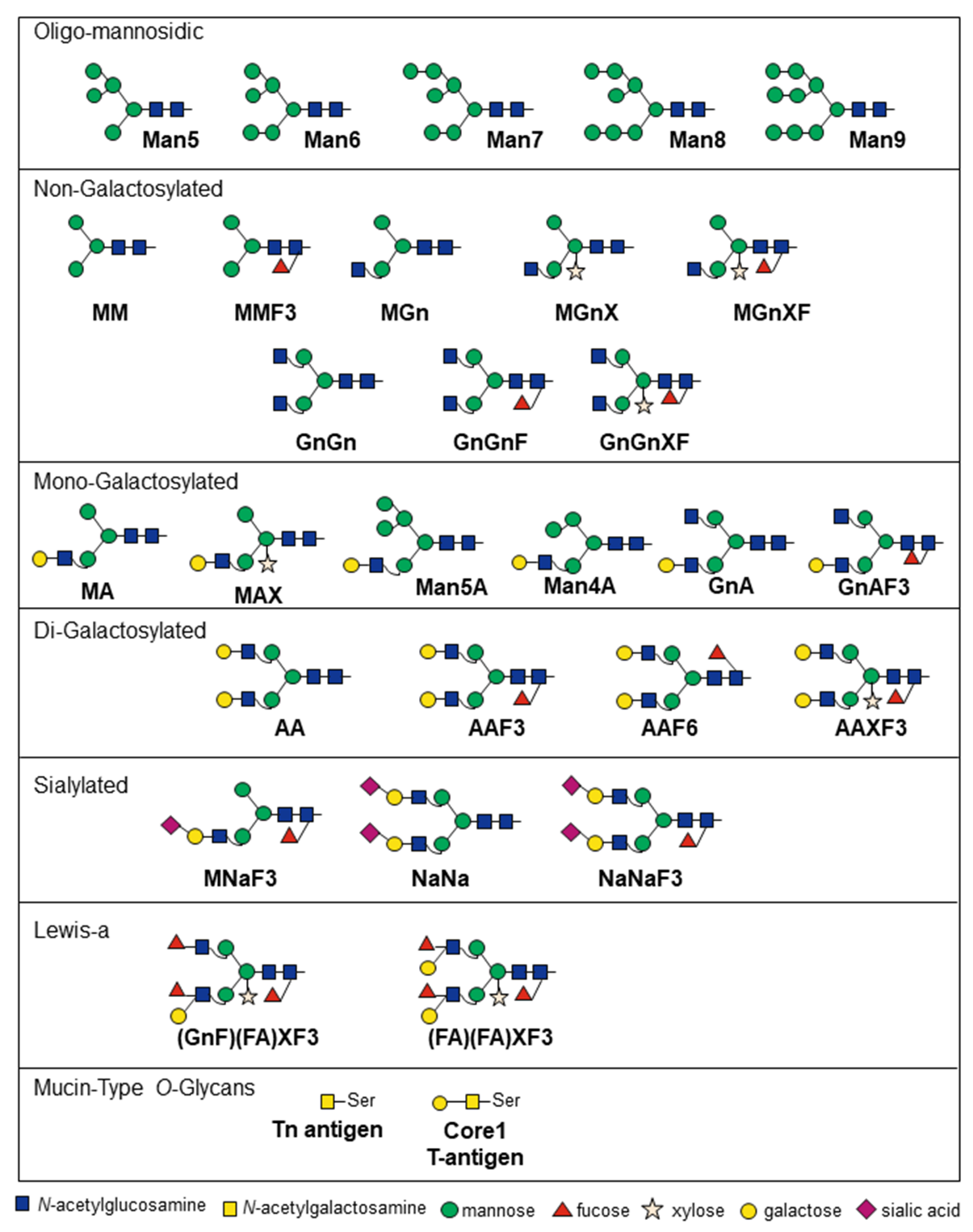
**

**Figure S13:** Schematic representation of the *N-* and *O*-glycan structures identify by LC-ESI-MS on recombinant glycoproteins expressed during this investigation. Glycan cartoons are in accordance to the Consortium for Functional Glycomics guidelines (www.functionalglycomics.org).
